# No influence of moon phases on emergency trauma admission

**DOI:** 10.1186/s13018-025-05778-0

**Published:** 2025-04-25

**Authors:** Filippo Migliorini, Nicola Maffulli, Marco Pilone, Ludovico Lucenti, Tommaso Bardazzi, Michael Memminger, Francesco Simeone, Gennaro Pipino, Christian David Weber

**Affiliations:** 1 Department of Trauma and Reconstructive Surgery, University Hospital of Halle, Martin-Luther University Halle-Wittenberg, 06097 Halle (Saale), Germany; 2 Department of Orthopaedic and Trauma Surgery, Academic Hospital of Bolzano (SABES-ASDAA), Via Lorenz Böhler 5, 39100 Bolzano, Italy; 3 Department of Life Sciences, Health, and Health Professions, Link Campus University, 00165 Rome, Italy; 4 Department of Orthopaedic and Trauma Surgery, Faculty of Medicine and Psychology, University La Sapienza, 00185 Rome, Italy; 5 School of Pharmacy and Bioengineering, Faculty of Medicine, Keele University, Stoke-on-Trent, ST4 7QB UK; 6 Centre for Sports and Exercise Medicine, Barts and the London School of Medicine and Dentistry, Mile End Hospital, Queen Mary University of London, London, E1 4DG UK; 7 Residency Program, University of Milan, Milan, Italy; 8 Department of Precision Medicine in Medical, Surgical and Critical Care (Me.Pre.C.C.), University of Palermo, 90133 Palermo, Italy; 9 Department of Orthopaedic Surgery, San Raffaele University, Milan, Italy; 10 Department of Orthopaedic, Trauma, and Reconstructive Surgery, RWTH University Medical Centre, 52074 Aachen, Germany

**Keywords:** Lunar, Moon, Phases, Trauma, Emergency department

## Abstract

**Introduction:**

The potential connection between lunar phases and human activities has fascinated scientists, medical professionals, and the general public. The present study evaluates the possible association between the moon phases and admissions to a major regional trauma hospital.

**Methods:**

All patients admitted to the trauma emergency department from 2018 to 2024 were retrieved. The Astronomical Applications Department of the U.S. Navy website was accessed to retrieve data on the lunar cycle phases. A multiple linear model regression analysis using the Pearson Product-Moment Correlation Coefficient (*r*) was used to assess the association between the number of patients admitted to the emergency trauma department and the percentage of the moon illuminated.

**Results:**

Data were collected from 53,594 patients (mean age was 36.0 ± 25.4 years); 45.4% (24,337 of 53,594) were women. There was no evidence of an association between the number of patients admitted to the trauma emergency department and moon phases (*P* = 0.1).

**Conclusion:**

There is no statistically significant association between moon phases and the frequency of visits to the trauma emergency department.

**Supplementary Information:**

The online version contains supplementary material available at 10.1186/s13018-025-05778-0.

## Introduction

The potential connection between lunar phases and human activities has fascinated scientists, medical professionals, and the broad community for ages [1, 2]. The idea of the moon’s effect on human behaviour, the"lunar effect", dates back to primitive societies [3, 4]. Ancient cultures often correlated the moon’s phases to some human events and health issues. They hypothesised an association given the visible effects of the moon on natural phenomena (such as tides); for this reason, they believed that comparable influences might occur within the human body [5–7]. Over the years, this hypothesis has grown, and most recent studies have tried to analytically examine the association between lunar phases and various aspects of human activity, such as crime rates, frequency of births, psychiatric disorders, and other events [3, 8, 9]. Lately, lunar phases, particularly the full moon, have been related to different occurrences, such as rising emergency department (ED) access, hospital admissions, and patient symptoms [1, 10, 11], with controversial findings [12–24]. An increased frequency of crimes and psychiatry admissions related to the moon cycle has been reported [2, 25]. Researchers have also studied the potential impact of lunar phases on other general medical emergencies, including cardiovascular, respiratory, and traumatic injuries [18]. Furthermore, a correlation between the distribution of spontaneous deliveries and the lunar month has been described mainly in multiparas and plurigravidas [26]. The moon’s gravitational pull might influence bodily fluid shifts and physiological processes, theoretically altering childbirth [27–30].

Despite these claims, scientific evidence remains inconclusive, and previous studies have yielded conflicting results. The primary objective of this study is to determine whether a statistical correlation exists between lunar phases and trauma-related emergency department admissions. This investigation is particularly relevant given the frequent references to the so-called"lunar effect"in scientific literature and popular belief. By analysing a large dataset spanning multiple years, this study aims to provide robust evidence to help clarify whether lunar phases influence the frequency of trauma-related emergency visits. According to some theories, the moon’s gravitational force may affect biological homeostasis in individuals. However, the exact foundation for this principle is vulnerable because of the relatively small gravitational forces implicated compared to other stronger influences on human physiology [31, 32]. Another explanation concerns psycho-social aspects**.** The cultural significance of the full moon may lead to heightened awareness and selective perception, potentially explaining the persistence of this belief despite a lack of strong empirical support [33, 34]. The primary challenge in studying the"lunar effect"lies in methodological inconsistencies. Variations in study design, sample size, and statistical analysis have led to conflicting results, making it difficult to draw clear conclusions. While some studies suggest a link between lunar phases and medical events, others do not, highlighting the need for large, well-structured studies to address these discrepancies [15, 35, 36]. Despite the controversy, understanding whether lunar phases affect trauma-related emergency admissions is important for emergency medicine. This study aims to fill that gap by providing robust statistical evidence to clarify the potential relationship. The present study evaluates the possible association between the moon phases and admissions to a major regional trauma hospital.

## Methods

### Study design

The present study was conducted according to the principles of the Declaration of Helsinki and approved by the ethics committee of the RWTH Aachen University (project ID EK 121/22). This investigation follows the Strengthening the Reporting of Observational Studies in Epidemiology: the STROBE Statement [37]. The present investigation was conducted at the Department of Orthopaedics, Trauma and Reconstructive Surgery of the University Hospital RWTH Aachen, Germany. In April 2024, the clinical databases of the institutions were accessed. For the databases of the German institutions, the OPS (operation and procedure codes) reported in Appendix 1 were used in combination with the ICD (International Statistical Classification of Diseases and Related Health Problems) codes, also noted in the appendix.

### Data collection

All patients admitted to the trauma ER from 2018 to 2024 were retrieved. The following data were retrieved: the number of patients admitted, the date of admission, and the age and gender of admitted patients. The Astronomical Applications Department of the U.S. Navy website [38] was accessed to retrieve data on the lunar cycle phases. The percentage of the moon illuminated each day was recorded independently by two authors (TB and FM) to reduce possible transcription mistakes. Data were collected in a Microsoft Excel spreadsheet (version 16.6, Microsoft Corporation, Redmond, USA). A Microsoft Excel spreadsheet was performed. Each row corresponds to a single day. Two columns were generated: one reporting the number of individuals accessing the emergency department and the other indicating the percentage of lunar illumination.

### Statistical analysis

All statistical analyses were performed by the main author (**) using the software STATA/MP 16.1 (StataCorp, College Station, TX, USA). For descriptive statistics, arithmetic mean and standard deviation were calculated. A multiple linear model regression analysis through the Pearson Product-Moment Correlation Coefficient (*r*) was used to assess the association between the number of patients admitted to the emergency trauma department and the percentage of the moon illuminated. The Cauchy–Schwarz formula was used for inequality: + 1 is considered a positive linear correlation, and − 1 is a negative one. Values of 0.1 <| *r* |< 0.3, 0.3 <| *r* |< 0.5, and | *r* |> 0.5 were considered to have a small, medium, and strong correlation, respectively. The overall significance was performed through the χ^2^ test, with values of P < 0.05 considered statistically significant. A linear regression model was then performed for the significant correlations. Added-variable plots were also performed for each comparison.

## Results

### Patient demographics

Data were collected from 53,594 patients, of which 45.4% (24,337 of 53,594) were females. The mean age was 36.0 ± 25.4 years. Demographic data are summarised in Table 1.

**Table 1:** Patient demographics

Patients (n)	53,594
Women	45.4% (24,337 of 53,594)
Mean age (*years*)	36.0 ± 25.4

### Result syntheses

**Fig. 1:**
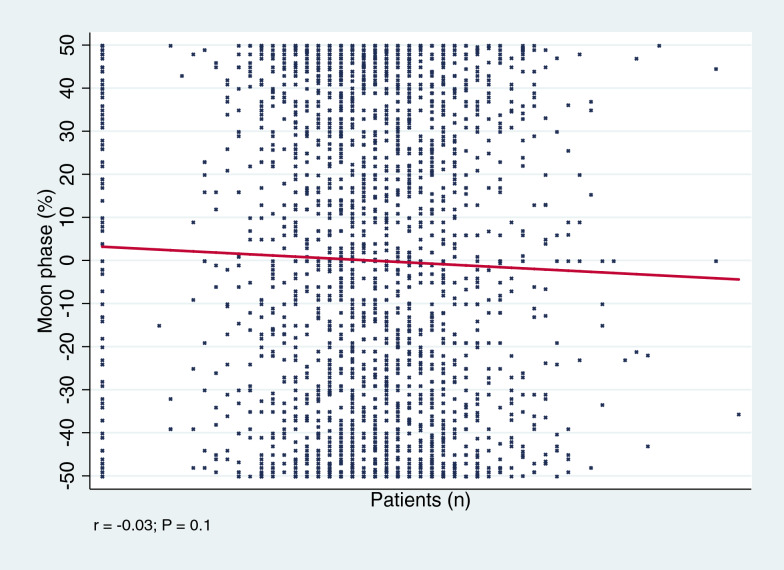
Added variable plot illustrating the results of a multiple linear regression analysis assessing the relationship between the number of patients admitted to the emergency trauma department and the percentage of lunar illumination. The y-axis represents the percentage of lunar illumination daily, while the x-axis reports the number of patients admitted to the emergency department daily. The red trend line expresses the association between the two variables, with a steeper slope indicating a stronger relationship

There was no evidence of an association between the number of patients admitted to the trauma emergency department and moon phases (*r* = −0.03; *P* = 0.1, Fig. 1).

## Discussion

The current study indicates no statistically significant association between the moon phases and the frequency of admissions to the trauma emergency department.

Throughout the centuries, lunar phases have captivated humanity, influencing beliefs, traditions, and, according to some, even health [3, 39, 40]. Myths and legends from various cultures speak of the moon’s influence on our well-being, tracing a delicate thread between the lunar cycle and human health [41–43]. In ancient Rome, individuals afflicted by illnesses and disorders would visit sanctuaries dedicated to Diana, hoping to receive her grace and healing power [44, 45]. The full moon’s light was believed to purify and invigorate the body and spirit, bringing relief and healing to those in need. Hippocrates’s humoral theory of health, postulated in the fifth century BC, shows that lunar phases influence the balance of bodily humours [46–48]. The full moon increased blood flow, the waning moon facilitated detoxification, the waxing moon promoted tissue regeneration, and the new moon heightened susceptibility to imbalances and illnesses. In traditional Chinese medicine, the moon phase is believed to influence various health aspects significantly. It is thought that body fluids, such as blood and Qi, are affected by the moon’s gravitational pull, leading to changes in circulation and fluid dynamics within the body. The full moon represents the peak of Yang energy, while the new moon represents the peak of Yin energy [49, 50]. In recent years, efforts to explore the potential link between lunar phases and emergency room visits have gained momentum [12, 40, 42, 51]. Researchers have undertaken studies analysing large datasets of emergency room admissions, seeking patterns or trends that coincide with lunar cycles [52]. In Yang et al.’s study [19], 559 cases of renal colic diagnoses at the University of Nebraska Medical Center were analysed over 24 months. The researchers compared these diagnoses with lunar phases and supermoon events [19]. No statistically significant association was found between the incidence of renal colic and lunar phases or supermoon events [19]. Akinpelu et al. [10] investigated the relationship between the lunar cycle and the prevalence and patterns of emergency urological presentation. 199 subjects were enrolled in the study, with no association between lunar phases and prevalence or patterns of emergency urological presentation. Saadat et al. [53] observed no association between moon phases and trauma death after having analysed 17,056 trauma deaths that occurred over ten years. Bunevicious et al. [42] examined the potential link between intracranial aneurysm rupture and the lunar cycle. Data from 1483 patients revealed no statistically significant association between lunar phases and aneurysm rupture [42]. However, one study in the review indicated a peak incidence of intracranial aneurysm rupture during the new moon phase [54]. Ruuskanen et al. [55] analysed and explored the potential correlation between the incidence rates of intracerebral haemorrhage (ICH) or ischemic stroke (IS) and lunar phases. They examined data from 94,894 IS patients and 17,855 ICH patients [55]. There was no statistically significant association between either admission or mortality rates and lunar phases in both groups [55]. Nardelli et al. [56] investigated the influence of moon phases on outcomes of total knee arthroplasty in 5923 patients, with no association between moon phases and post-operative functional scores or revision rate [56]. Ficklscherer et al. [57] analysed the influence of the lunar phases on perioperative complications in 305 patients who underwent total hip arthroplasty. Data on the possible influences of lunar phases on perioperative complications, such as operation length, blood loss, and course of C-reactive protein, were collected to identify potential influences of lunar phases on perioperative complications [57]. No association was found between perioperative complications and lunar phases [57].

The moon’s phases, specific dates, and zodiac signs are merely objects of mystical superstition, lacking any scientific foundation. Our ancestors turned to them in an attempt to explain phenomena that they were unable to comprehend. Across centuries, myths and legends have intertwined with reality, shaping modern superstitions. Even events documented in history have influenced present-day beliefs. For example, some believe that Friday the 13 th brings bad luck. In 1307, Philip IV ordered the arrest of all Knights Templar.

Compared with other published material, this study contributes to the field by analysing a substantially larger dataset over multiple years with a robust statistical approach. While previous studies have often focused on specific medical conditions or psychiatric admissions, our research examines trauma-related emergency visits, an area where evidence remains sparse. This study contributes to the field by analysing a substantially larger dataset with a robust statistical approach over multiple years. While previous research has primarily focused on psychiatric admissions or specific medical conditions, our study examines trauma-related emergency visits, an area where evidence remains sparse. By employing a methodologically rigorous approach with a large and diverse patient population, this investigation strengthens the body of literature debunking the so-called lunar effect in emergency medicine. The strengths of this study lie in its extensive sample size and prolonged study period, which enhance the reliability and generalizability of the findings. A robust statistical model also ensures high methodological accuracy, reinforcing the conclusion that lunar phases do not influence trauma-related emergency department admissions. These findings contribute valuable evidence to emergency medicine by dispelling a persistent myth while providing data-driven insights that can help optimise hospital resource planning based on actual trends rather than unfounded lunar cycle assumptions.

This study has multiple limitations. It is a retrospective, single-centre study conducted over a limited period. Additionally, the patients’ comorbidities were not taken into account. Common limitations in all medical record review studies include the potential for spurious, incomplete or conflicting data. Naturally, constraints are associated with variations in the Earth’s axial tilt and disruptions in the lunar orbit caused by gravitational influences from other celestial bodies, which were not addressed in this study. The lack of adjustment for potential confounding factors such as seasonal variations, public holidays, and specific high-risk time frames (e.g., weekends) also represents another limitation of the present study. The causes of trauma, related severity and outcomes (such as discharge, hospitalisation or death) were not considered. Future research could refine the analysis by integrating additional variables influencing emergency department admissions, such as meteorological conditions, alcohol consumption patterns, and socio-economic fluctuations. A multicentric approach across different geographic areas could provide more generalisable results.

## Conclusion

No statistically significant association was found between the moon phases and the frequency of visits to the trauma emergency department.

## Supplementary Information

Supplementary file 1. 

## Data Availability

No datasets were generated or analysed during the current study.
