# Supplementary material for: No influence of moon phases on emergency trauma admission
Source: J Orthop Surg Res. 2025 Apr 25;20:417. doi: 10.1186/s13018-025-05778-0 (PMC12023559; doi:10.1186/s13018-025-05778-0)
Supplement: Supplementary file 1 [file 13018_2025_5778_MOESM1_ESM.docx]

**OPS:**

1-100, 1-202.00, 1-202.01, 1-202.1, 1-204.0, 1-204.1, 1-204.2, 1-204.5, 1-205, 1-206, 1-207.0, 1-207.1, 1-207.2, 1-207.3, 1-208.1, 1-208.2, 1-208.3, 1-208.4, 1-208.6, 1-208.8, 1-210, 1-220.0, 1-242, 1-243, 1-265.1, 1-265.4, 1-265.5, 1-265.6, 1-265.8, 1-265.f, 1-266.0, 1-266.1, 1-266.2, 1-268.0, 1-268.1, 1-268.3, 1-273.1, 1-273.2, 1-273.6, 1-274.3, 1-274.30, 1-275.0, 1-275.1, 1-275.2, 1-275.5, 1-276.1, 1-276.21, 1-279.0, 1-279.1, 1-279.a, 1-334.1, 1-415, 1-424, 1-425.3, 1-426.3, 1-426.4, 1-426.6, 1-430.10, 1-430.x, 1-432.01, 1-440.9, 1-440.a, 1-442.0, 1-444.6, 1-444.7, 1-447, 1-463.0, 1-465.0, 1-465.8, 1-472.0, 1-480.4, 1-480.6, 1-481.4, 1-481.5, 1-482.1, 1-482.7, 1-490.0, 1-490.1, 1-490.2, 1-490.3, 1-490.4, 1-490.5, 1-490.6, 1-490.7, 1-492.1, 1-492.4, 1-492.5, 1-493.30, 1-493.31, 1-494.30, 1-494.8, 1-494.x, 1-503.1, 1-503.4, 1-503.5, 1-503.6, 1-503.7, 1-503.8, 1-504.1, 1-504.6, 1-504.7, 1-510.0, 1-511.00, 1-513.8, 1-531, 1-581.0, 1-610.0, 1-610.1, 1-610.2, 1-610.x, 1-611.0, 1-611.00, 1-611.1, 1-611.x, 1-612, 1-613, 1-620.00, 1-620.01, 1-620.0x, 1-620.10, 1-620.1x, 1-620.x, 1-630.0, 1-630.1, 1-631.0, 1-632.0, 1-632.1, 1-632.x, 1-636.1, 1-640, 1-641, 1-642, 1-650.0, 1-650.1, 1-650.2, 1-651, 1-652.0, 1-652.1, 1-652.2, 1-653, 1-654.0, 1-654.1, 1-654.x, 1-660, 1-661, 1-668.1, 1-672, 1-691.0, 1-693.2, 1-694, 1-695.x, 1-697.1, 1-697.6, 1-697.7, 1-710, 1-712, 1-717.0, 1-717.1, 1-760, 1-774, 1-842, 1-843, 1-844, 1-850, 1-853.0, 1-853.2, 1-854.1, 1-854.2, 1-854.6, 1-854.7, 1-854.8, 1-854.a, 1-859.x, 1-930.0, 1-931.1, 1-941.0, 1-991.3, 1-999.42, 3-030, 3-034, 3-035, 3-052, 3-053, 3-054, 3-055, 3-055.0, 3-055.1, 3-056, 3-059, 3-05a, 3-05f, 3-05g.0, 3-100.0, 3-13c.1, 3-13c.3, 3-13d.5, 3-13d.6, 3-13f, 3-13g, 3-200, 3-201, 3-202, 3-203, 3-205, 3-206, 3-207, 3-208, 3-20x, 3-220, 3-221, 3-222, 3-223, 3-224.30, 3-225, 3-226, 3-227, 3-228, 3-22x, 3-241, 3-24x, 3-600, 3-601, 3-603, 3-604, 3-605, 3-606, 3-607, 3-608, 3-60a, 3-60x, 3-611.0, 3-611.x, 3-612.0, 3-612.2, 3-612.4, 3-612.5, 3-614, 3-701, 3-703.0, 3-703.1, 3-705.0, 3-705.1, 3-706.1, 3-720.10, 3-721.01, 3-721.11, 3-721.21, 3-722.1, 3-724.0, 3-732.0, 3-733.0, 3-740, 3-752.0, 3-752.1, 3-753.0, 3-753.1, 3-800, 3-801, 3-802, 3-803.0, 3-804, 3-805, 3-806, 3-808, 3-809, 3-820, 3-821, 3-823, 3-824.0, 3-824.2, 3-825, 3-826, 3-828, 3-82a, 3-84x, 3-900, 3-990, 3-991, 3-992, 3-993, 3-994, 3-995, 3-996, 5-010.00, 5-010.01, 5-010.02, 5-010.03, 5-010.04, 5-010.10, 5-010.11, 5-010.13, 5-010.14, 5-010.2, 5-011.0, 5-012.0, 5-012.2, 5-012.4, 5-012.5, 5-012.x, 5-013.0, 5-013.1, 5-013.2, 5-013.4, 5-013.40, 5-013.50, 5-013.6, 5-013.8, 5-013.x, 5-015.0, 5-015.1, 5-015.20, 5-015.x, 5-016.3, 5-020.10, 5-020.11, 5-020.12, 5-020.2, 5-020.3, 5-020.4, 5-020.60, 5-020.61, 5-020.70, 5-021.0, 5-021.1, 5-021.2, 5-021.3, 5-021.4, 5-021.6, 5-022.00, 5-023.10, 5-023.12, 5-024.0, 5-024.6, 5-024.7, 5-024.x, 5-025.5, 5-029.10, 5-029.11, 5-029.12, 5-029.c, 5-029.x, 5-030.1, 5-030.2, 5-030.30, 5-030.31, 5-030.32, 5-030.40, 5-030.41, 5-030.51, 5-030.52, 5-030.60, 5-030.61, 5-030.62, 5-030.70, 5-030.71, 5-030.72, 5-030.8, 5-030.x, 5-031.00, 5-031.01, 5-031.02, 5-031.10, 5-031.11, 5-031.20, 5-031.21, 5-031.22, 5-031.30, 5-031.31, 5-031.32, 5-031.5, 5-031.6, 5-031.7, 5-031.8, 5-031.x, 5-032.00, 5-032.01, 5-032.02, 5-032.10, 5-032.11, 5-032.20, 5-032.21, 5-032.30, 5-032.31, 5-032.40, 5-032.41, 5-032.42, 5-032.5, 5-032.6, 5-032.7, 5-032.8, 5-032.9, 5-032.b, 5-032.c, 5-032.x, 5-033.0, 5-033.2, 5-033.3, 5-033.4, 5-034.1, 5-034.5, 5-034.6, 5-035.2, 5-035.7, 5-036.5, 5-036.6, 5-036.8, 5-037.0, 5-037.2, 5-037.x, 5-038.0, 5-039.0, 5-039.a4, 5-041.3, 5-041.8, 5-042.0, 5-042.1, 5-042.x, 5-044.1, 5-044.3, 5-044.4, 5-044.5, 5-044.8, 5-045.3, 5-045.4, 5-045.8, 5-046.3, 5-046.4, 5-047.8, 5-048.1, 5-048.3, 5-048.y, 5-049.y, 5-04b.0, 5-04b.1, 5-04b.3, 5-04b.4, 5-04b.8, 5-050.8, 5-055.8, 5-056.0, 5-056.1, 5-056.3, 5-056.40, 5-056.5, 5-056.8, 5-056.9, 5-057.3, 5-058.43, 5-059.b, 5-062.0, 5-078.x, 5-086.1, 5-086.30, 5-086.31, 5-093.1, 5-095.00, 5-095.01, 5-095.10, 5-095.11, 5-096.50, 5-096.51, 5-10e.2, 5-10e.3, 5-110.x, 5-115, 5-124, 5-136.3, 5-137.1, 5-137.x, 5-138.10, 5-139.0, 5-139.10, 5-145.20, 5-145.2k, 5-154.0, 5-154.2, 5-155.7, 5-156.1, 5-158.11, 5-158.13, 5-158.33, 5-158.43, 5-160.2, 5-160.3, 5-160.x, 5-166.04, 5-166.1, 5-166.2, 5-166.3, 5-167.1, 5-167.2, 5-168.x, 5-169.0x, 5-180.0, 5-180.2, 5-180.3, 5-181.0, 5-181.1, 5-181.2, 5-183.0, 5-183.1, 5-183.y, 5-185.2, 5-195.b2, 5-195.b3, 5-200.5, 5-202.2, 5-203.0, 5-203.71, 5-204.2, 5-210.1, 5-211.11, 5-214.0, 5-214.5, 5-214.6, 5-215.1, 5-216.0, 5-216.1, 5-216.2, 5-216.x, 5-217.1, 5-217.3, 5-218.40, 5-218.4x, 5-219.x, 5-224.63, 5-225.5, 5-230.0, 5-230.1, 5-230.2, 5-230.3, 5-230.5, 5-231.00, 5-231.03, 5-231.10, 5-231.20, 5-231.50, 5-231.x3, 5-232.02, 5-232.x0, 5-235.0, 5-235.5, 5-235.6, 5-235.7, 5-237.0, 5-240.2, 5-241.0, 5-242.4, 5-243.3, 5-249.0, 5-249.x, 5-250.0, 5-253.0, 5-253.1, 5-270.0, 5-270.1, 5-270.3, 5-270.6, 5-273.3, 5-273.90, 5-273.x, 5-274.0, 5-275.0, 5-279.0, 5-280.1, 5-292.0, 5-292.30, 5-294.1, 5-311.0, 5-311.1, 5-311.3, 5-312.0, 5-312.1, 5-312.2, 5-312.x, 5-313.3, 5-314.00, 5-315.0, 5-315.2, 5-315.5, 5-316.0, 5-316.2, 5-316.3, 5-316.x, 5-319.x, 5-322.d1, 5-322.e4, 5-322.g1, 5-324.a1, 5-328.01, 5-333.0, 5-333.1, 5-334.0, 5-334.1, 5-334.2, 5-334.7, 5-334.8, 5-339.x, 5-340.0, 5-340.1, 5-340.5, 5-340.a, 5-340.b, 5-340.c, 5-340.d, 5-340.x, 5-341.0, 5-341.1, 5-342.01, 5-342.03, 5-343.0, 5-343.1, 5-343.2, 5-343.4, 5-343.7, 5-343.x, 5-344.0, 5-344.10, 5-344.12, 5-344.3, 5-344.40, 5-344.42, 5-346.4, 5-346.5, 5-346.c0, 5-346.c1, 5-346.c2, 5-346.c3, 5-346.cx, 5-346.d1, 5-346.d2, 5-346.x, 5-347.1, 5-347.2, 5-347.30, 5-349.0, 5-349.6, 5-35a.05, 5-35b.11, 5-361.03, 5-361.07, 5-361.17, 5-362.03, 5-364.00, 5-370.0, 5-370.1, 5-370.3, 5-372.0, 5-374.3, 5-374.x, 5-377.1, 5-377.30, 5-377.31, 5-377.50, 5-377.6, 5-377.8, 5-377.c0, 5-378.18, 5-378.52, 5-378.b4, 5-379.0, 5-37b.00, 5-37b.01, 5-37b.10, 5-37b.32, 5-380.0x, 5-380.12, 5-380.20, 5-380.23, 5-380.24, 5-380.2x, 5-380.31, 5-380.4x, 5-380.53, 5-380.54, 5-380.56, 5-380.70, 5-380.71, 5-380.72, 5-380.73, 5-380.80, 5-380.83, 5-380.84, 5-380.97, 5-380.98, 5-380.99, 5-380.9a, 5-380.9b, 5-380.9c, 5-380.9x, 5-380.a6, 5-381.01, 5-381.02, 5-381.03, 5-381.54, 5-381.70, 5-381.71, 5-381.72, 5-382.01, 5-382.12, 5-382.24, 5-382.72, 5-382.a1, 5-382.a6, 5-383.24, 5-383.70, 5-383.72, 5-383.9b, 5-383.9c, 5-383.a6, 5-384.73, 5-384.76, 5-385.1, 5-385.72, 5-386.a2, 5-386.a5, 5-386.a6, 5-388.01, 5-388.02, 5-388.03, 5-388.04, 5-388.0x, 5-388.12, 5-388.20, 5-388.23, 5-388.24, 5-388.2x, 5-388.32, 5-388.40, 5-388.53, 5-388.54, 5-388.55, 5-388.64, 5-388.6x, 5-388.70, 5-388.71, 5-388.72, 5-388.7x, 5-388.80, 5-388.83, 5-388.91, 5-388.96, 5-388.97, 5-388.99, 5-388.9b, 5-388.9g, 5-388.9x, 5-388.a0, 5-388.a2, 5-388.a4, 5-388.a6, 5-388.x, 5-388.y, 5-389.03, 5-389.04, 5-389.0x, 5-389.23, 5-389.24, 5-389.2x, 5-389.32, 5-389.4x, 5-389.5x, 5-389.70, 5-389.82, 5-389.83, 5-389.8x, 5-389.91, 5-389.94, 5-389.98, 5-389.99, 5-389.9b, 5-389.9c, 5-389.9k, 5-389.9x, 5-389.a1, 5-389.a6, 5-389.x, 5-38a.70, 5-38a.7x, 5-38a.a0, 5-38a.c0, 5-38a.u0, 5-38a.u1, 5-392.10, 5-392.11, 5-392.70, 5-393.01, 5-393.02, 5-393.2, 5-393.42, 5-393.51, 5-393.52, 5-393.54, 5-393.61, 5-393.7, 5-394.0, 5-394.1, 5-394.2, 5-394.4, 5-394.5, 5-394.8, 5-394.90, 5-395.40, 5-395.70, 5-395.71, 5-395.80, 5-395.a1, 5-397.12, 5-397.24, 5-397.27, 5-397.7x, 5-399.2, 5-399.3, 5-399.5, 5-399.7, 5-401.10, 5-401.50, 5-401.b, 5-402.0, 5-406.2, 5-406.3, 5-406.a, 5-407.2, 5-413.00, 5-413.10, 5-413.12, 5-419.0, 5-419.3, 5-419.x, 5-429.40, 5-429.d, 5-429.u, 5-431.20, 5-431.21, 5-433.42, 5-433.50, 5-434.0, 5-448.00, 5-448.02, 5-449.x2, 5-450.1, 5-450.3, 5-452.61, 5-454.12, 5-454.20, 5-454.30, 5-454.40, 5-454.60, 5-454.62, 5-455.27, 5-455.2x, 5-455.41, 5-455.42, 5-455.44, 5-455.47, 5-455.62, 5-455.71, 5-455.72, 5-455.76, 5-455.77, 5-455.7x, 5-455.a2, 5-456.00, 5-459.0, 5-460.10, 5-460.11, 5-460.40, 5-460.41, 5-460.51, 5-461.10, 5-461.41, 5-461.50, 5-462.0, 5-462.1, 5-462.4, 5-462.5, 5-462.6, 5-463.21, 5-464.21, 5-464.22, 5-465.1, 5-466.2, 5-467.00, 5-467.01, 5-467.02, 5-467.03, 5-467.0x, 5-467.11, 5-467.x1, 5-469.00, 5-469.03, 5-469.11, 5-469.20, 5-469.21, 5-469.70, 5-469.72, 5-469.e3, 5-469.k3, 5-469.x2, 5-470.0, 5-470.10, 5-470.11, 5-484.32, 5-484.39, 5-486.0, 5-490.1, 5-496.0, 5-496.3, 5-501.01, 5-501.20, 5-502.0, 5-502.2, 5-505.0, 5-505.1, 5-505.x, 5-509.x, 5-511.01, 5-511.11, 5-511.41, 5-512.40, 5-513.1, 5-513.21, 5-513.42, 5-513.b, 5-513.f0, 5-514.50, 5-514.53, 5-514.d0, 5-514.n3, 5-514.p3, 5-516.0, 5-519.x, 5-524.00, 5-526.e1, 5-530.31, 5-534.03, 5-535.0, 5-535.3x, 5-536.0, 5-539.0, 5-540.0, 5-541.0, 5-541.1, 5-541.2, 5-541.3, 5-541.4, 5-541.5, 5-542.0, 5-543.y, 5-545.1, 5-546.0, 5-546.1, 5-546.21, 5-546.22, 5-549.0, 5-549.5, 5-549.x, 5-550.1, 5-554.40, 5-554.a1, 5-557.02, 5-562.4, 5-562.5, 5-562.9, 5-568.00, 5-570.0, 5-570.4, 5-572.1, 5-572.5, 5-573.32, 5-573.40, 5-576.20, 5-578.00, 5-578.0x, 5-584.x, 5-585.0, 5-590.20, 5-590.2x, 5-590.80, 5-591.0, 5-601.x, 5-612.1, 5-622.0, 5-640.2, 5-642.0, 5-651.92, 5-653.32, 5-671.00, 5-681.80, 5-681.x, 5-690.1, 5-706.0, 5-710, 5-716.0, 5-740.1, 5-758.4, 5-760.13, 5-760.14, 5-760.23, 5-760.24, 5-760.3, 5-760.4x, 5-760.63, 5-760.x3, 5-761.12, 5-761.13, 5-761.14, 5-761.2, 5-761.33, 5-761.43, 5-762.13, 5-762.x3, 5-763.13, 5-763.33, 5-763.53, 5-763.73, 5-764.13, 5-764.14, 5-764.20, 5-764.22, 5-764.23, 5-764.24, 5-764.43, 5-764.x3, 5-765.13, 5-765.23, 5-765.33, 5-765.34, 5-765.43, 5-765.63, 5-765.70, 5-765.73, 5-766.0, 5-766.1, 5-766.2, 5-766.3, 5-766.4, 5-766.5, 5-766.6, 5-767.0, 5-767.3, 5-767.4, 5-769.0, 5-769.1, 5-769.2, 5-769.4, 5-769.6, 5-770.4, 5-770.7, 5-770.8, 5-773.9, 5-778.0, 5-778.2, 5-779.0, 5-779.3, 5-779.4, 5-780.07, 5-780.0f, 5-780.0k, 5-780.12, 5-780.14, 5-780.1f, 5-780.1g, 5-780.1h, 5-780.1k, 5-780.1m, 5-780.1n, 5-780.1s, 5-780.1t, 5-780.2d, 5-780.2t, 5-780.36, 5-780.3h, 5-780.3k, 5-780.42, 5-780.46, 5-780.48, 5-780.4d, 5-780.4e, 5-780.4f, 5-780.4g, 5-780.4h, 5-780.4k, 5-780.4m, 5-780.4n, 5-780.4t, 5-780.4x, 5-780.5d, 5-780.5f, 5-780.5g, 5-780.61, 5-780.62, 5-780.63, 5-780.64, 5-780.65, 5-780.66, 5-780.67, 5-780.68, 5-780.69, 5-780.6a, 5-780.6b, 5-780.6c, 5-780.6d, 5-780.6f, 5-780.6g, 5-780.6h, 5-780.6j, 5-780.6k, 5-780.6m, 5-780.6n, 5-780.6p, 5-780.6q, 5-780.6r, 5-780.6s, 5-780.6t, 5-780.6v, 5-780.6w, 5-780.6x, 5-780.7h, 5-780.7m, 5-780.8g, 5-780.8u, 5-780.9d, 5-780.9f, 5-780.9k, 5-781.16, 5-781.1h, 5-781.39, 5-781.3m, 5-781.3q, 5-781.7g, 5-781.a6, 5-781.a7, 5-781.af, 5-781.ag, 5-781.an, 5-782.11, 5-782.13, 5-782.1d, 5-782.1e, 5-782.1g, 5-782.1k, 5-782.1m, 5-782.2e, 5-782.2f, 5-782.2m, 5-782.2r, 5-782.2u, 5-782.31, 5-782.44, 5-782.4f, 5-782.4r, 5-782.5f, 5-782.5g, 5-782.6c, 5-782.6g, 5-782.6q, 5-782.6x, 5-782.9g, 5-782.a0, 5-782.a2, 5-782.a3, 5-782.a6, 5-782.a7, 5-782.a9, 5-782.ad, 5-782.ae, 5-782.af, 5-782.ag, 5-782.ah, 5-782.ak, 5-782.am, 5-782.an, 5-782.ar, 5-782.as, 5-782.at, 5-782.az, 5-782.xm, 5-782.xr, 5-782.xu, 5-783.06, 5-783.0d, 5-783.0s, 5-783.0x, 5-783.2d, 5-783.7q, 5-783.xx, 5-784.01, 5-784.02, 5-784.07, 5-784.0a, 5-784.0d, 5-784.0e, 5-784.0f, 5-784.0g, 5-784.0h, 5-784.0k, 5-784.0n, 5-784.0s, 5-784.0t, 5-784.17, 5-784.19, 5-784.1d, 5-784.1k, 5-784.1n, 5-784.2f, 5-784.2u, 5-784.2v, 5-784.4m, 5-784.4q, 5-784.6n, 5-784.70, 5-784.71, 5-784.76, 5-784.7d, 5-784.7e, 5-784.7f, 5-784.7g, 5-784.7h, 5-784.7k, 5-784.7n, 5-784.7r, 5-784.7s, 5-784.7t, 5-784.81, 5-784.82, 5-784.83, 5-784.8d, 5-784.8h, 5-784.bh, 5-784.x1, 5-784.x2, 5-784.y, 5-785.01, 5-785.02, 5-785.03, 5-785.0e, 5-785.0f, 5-785.0h, 5-785.0x, 5-785.11, 5-785.12, 5-785.1d, 5-785.1e, 5-785.1f, 5-785.1g, 5-785.1k, 5-785.1m, 5-785.1n, 5-785.20, 5-785.21, 5-785.23, 5-785.25, 5-785.26, 5-785.28, 5-785.29, 5-785.2d, 5-785.2f, 5-785.2h, 5-785.2k, 5-785.2m, 5-785.2n, 5-785.2s, 5-785.2t, 5-785.30, 5-785.31, 5-785.32, 5-785.33, 5-785.35, 5-785.36, 5-785.37, 5-785.3e, 5-785.3f, 5-785.3g, 5-785.3h, 5-785.3j, 5-785.3k, 5-785.3m, 5-785.3n, 5-785.3r, 5-785.3s, 5-785.3t, 5-785.3u, 5-785.4d, 5-785.5d, 5-785.5e, 5-785.5f, 5-785.5k, 5-785.5n, 5-785.5u, 5-785.62, 5-785.6e, 5-785.6k, 5-785.7k, 5-785.y, 5-786.0, 5-786.1, 5-786.2, 5-786.3, 5-786.4, 5-786.5, 5-786.6, 5-786.7, 5-786.8, 5-786.c, 5-786.e, 5-786.g, 5-786.k, 5-786.p, 5-786.x, 5-787.01, 5-787.03, 5-787.04, 5-787.06, 5-787.07, 5-787.08, 5-787.09, 5-787.0a, 5-787.0b, 5-787.0c, 5-787.0d, 5-787.0f, 5-787.0g, 5-787.0h, 5-787.0k, 5-787.0n, 5-787.0t, 5-787.0u, 5-787.0v, 5-787.0w, 5-787.0x, 5-787.11, 5-787.13, 5-787.14, 5-787.16, 5-787.17, 5-787.19, 5-787.1b, 5-787.1d, 5-787.1e, 5-787.1f, 5-787.1g, 5-787.1h, 5-787.1j, 5-787.1k, 5-787.1m, 5-787.1n, 5-787.1q, 5-787.1r, 5-787.1s, 5-787.1t, 5-787.1u, 5-787.1v, 5-787.23, 5-787.2d, 5-787.2e, 5-787.2f, 5-787.2g, 5-787.2h, 5-787.2j, 5-787.31, 5-787.32, 5-787.33, 5-787.35, 5-787.37, 5-787.38, 5-787.39, 5-787.3d, 5-787.3e, 5-787.3f, 5-787.3g, 5-787.3h, 5-787.3k, 5-787.3m, 5-787.3n, 5-787.3r, 5-787.3t, 5-787.3w, 5-787.43, 5-787.4h, 5-787.5e, 5-787.5f, 5-787.6f, 5-787.6g, 5-787.6m, 5-787.6n, 5-787.7f, 5-787.7g, 5-787.8m, 5-787.91, 5-787.92, 5-787.93, 5-787.95, 5-787.96, 5-787.97, 5-787.98, 5-787.9b, 5-787.9d, 5-787.9e, 5-787.9f, 5-787.9g, 5-787.9h, 5-787.9k, 5-787.9m, 5-787.9n, 5-787.9r, 5-787.9s, 5-787.9t, 5-787.9u, 5-787.9v, 5-787.9x, 5-787.cn, 5-787.g2, 5-787.g5, 5-787.g8, 5-787.g9, 5-787.k1, 5-787.k2, 5-787.k3, 5-787.k5, 5-787.k6, 5-787.k8, 5-787.k9, 5-787.kf, 5-787.kg, 5-787.kh, 5-787.kk, 5-787.km, 5-787.kn, 5-787.kr, 5-787.kt, 5-787.ku, 5-787.m3, 5-787.mg, 5-787.mm, 5-787.mn, 5-787.n4, 5-787.ps, 5-787.pu, 5-787.pv, 5-787.xf, 5-787.xg, 5-787.xh, 5-787.xk, 5-787.xx, 5-788.65, 5-789.01, 5-789.0j, 5-789.0x, 5-789.1d, 5-789.2e, 5-789.31, 5-789.34, 5-789.3d, 5-789.3e, 5-789.3g, 5-789.3k, 5-789.3m, 5-789.3v, 5-789.be, 5-789.bf, 5-789.bg, 5-789.bh, 5-789.xm, 5-78a.01, 5-78a.06, 5-78a.07, 5-78a.0d, 5-78a.0e, 5-78a.0f, 5-78a.0g, 5-78a.0k, 5-78a.0n, 5-78a.0t, 5-78a.0v, 5-78a.11, 5-78a.1f, 5-78a.1g, 5-78a.1h, 5-78a.1n, 5-78a.1t, 5-78a.1w, 5-78a.25, 5-78a.2t, 5-78a.3f, 5-78a.5f, 5-78a.6f, 5-78a.6g, 5-78a.7m, 5-78a.8g, 5-78a.8k, 5-78a.8m, 5-78a.8n, 5-78a.g2, 5-78a.k3, 5-78a.k9, 5-78a.kg, 5-78a.kr, 5-78a.mk, 5-78a.mm, 5-78a.xf, 5-790.01, 5-790.02, 5-790.06, 5-790.08, 5-790.0a, 5-790.0c, 5-790.0d, 5-790.0e, 5-790.0f, 5-790.0g, 5-790.0h, 5-790.0j, 5-790.0k, 5-790.0m, 5-790.0n, 5-790.0r, 5-790.0s, 5-790.0t, 5-790.0u, 5-790.0z, 5-790.10, 5-790.11, 5-790.12, 5-790.13, 5-790.14, 5-790.15, 5-790.16, 5-790.17, 5-790.18, 5-790.19, 5-790.1a, 5-790.1b, 5-790.1c, 5-790.1f, 5-790.1g, 5-790.1h, 5-790.1k, 5-790.1m, 5-790.1n, 5-790.1r, 5-790.1s, 5-790.1t, 5-790.1u, 5-790.1v, 5-790.1w, 5-790.1x, 5-790.21, 5-790.22, 5-790.23, 5-790.24, 5-790.25, 5-790.26, 5-790.27, 5-790.28, 5-790.29, 5-790.2b, 5-790.2c, 5-790.2f, 5-790.2g, 5-790.2k, 5-790.2m, 5-790.2q, 5-790.2r, 5-790.2v, 5-790.2w, 5-790.31, 5-790.32, 5-790.3e, 5-790.3f, 5-790.3g, 5-790.3h, 5-790.3k, 5-790.3m, 5-790.3n, 5-790.3r, 5-790.42, 5-790.4f, 5-790.4g, 5-790.4h, 5-790.4m, 5-790.4r, 5-790.58, 5-790.5e, 5-790.5f, 5-790.5g, 5-790.5h, 5-790.5m, 5-790.62, 5-790.63, 5-790.64, 5-790.65, 5-790.66, 5-790.67, 5-790.68, 5-790.69, 5-790.6a, 5-790.6d, 5-790.6e, 5-790.6f, 5-790.6g, 5-790.6h, 5-790.6j, 5-790.6k, 5-790.6m, 5-790.6n, 5-790.6p, 5-790.6q, 5-790.6r, 5-790.6s, 5-790.6t, 5-790.6u, 5-790.6v, 5-790.6x, 5-790.7k, 5-790.7n, 5-790.8e, 5-790.8f, 5-790.k6, 5-790.k8, 5-790.k9, 5-790.kd, 5-790.kh, 5-790.kk, 5-790.kn, 5-790.mk, 5-790.mm, 5-790.n1, 5-790.ne, 5-790.nh, 5-790.nm, 5-790.nr, 5-790.p3, 5-790.pc, 5-790.pd, 5-790.pn, 5-790.qd, 5-790.x2, 5-790.xd, 5-791.02, 5-791.05, 5-791.08, 5-791.0g, 5-791.0m, 5-791.0q, 5-791.15, 5-791.18, 5-791.1g, 5-791.1m, 5-791.22, 5-791.25, 5-791.28, 5-791.2g, 5-791.2m, 5-791.2q, 5-791.2x, 5-791.5g, 5-791.6g, 5-791.6m, 5-791.7g, 5-791.7m, 5-791.7q, 5-791.82, 5-791.8g, 5-791.8m, 5-791.g2, 5-791.g5, 5-791.g8, 5-791.gm, 5-791.gq, 5-791.k2, 5-791.k5, 5-791.k8, 5-791.kg, 5-791.km, 5-791.kq, 5-791.kx, 5-791.ng, 5-792.02, 5-792.05, 5-792.08, 5-792.0g, 5-792.0m, 5-792.0q, 5-792.12, 5-792.15, 5-792.18, 5-792.1g, 5-792.1q, 5-792.22, 5-792.25, 5-792.28, 5-792.2g, 5-792.2m, 5-792.2q, 5-792.3g, 5-792.5g, 5-792.6g, 5-792.6m, 5-792.6q, 5-792.7g, 5-792.7m, 5-792.85, 5-792.88, 5-792.8g, 5-792.8m, 5-792.g5, 5-792.g8, 5-792.k2, 5-792.k5, 5-792.k8, 5-792.kg, 5-792.km, 5-792.kq, 5-792.m5, 5-792.m8, 5-792.mm, 5-792.nx, 5-793.0f, 5-793.0n, 5-793.0r, 5-793.11, 5-793.13, 5-793.14, 5-793.16, 5-793.17, 5-793.19, 5-793.1e, 5-793.1f, 5-793.1h, 5-793.1j, 5-793.1k, 5-793.1n, 5-793.1p, 5-793.1r, 5-793.21, 5-793.23, 5-793.24, 5-793.26, 5-793.27, 5-793.29, 5-793.2e, 5-793.2f, 5-793.2h, 5-793.2j, 5-793.2k, 5-793.2n, 5-793.2r, 5-793.31, 5-793.33, 5-793.34, 5-793.36, 5-793.37, 5-793.39, 5-793.3e, 5-793.3f, 5-793.3h, 5-793.3k, 5-793.3n, 5-793.3r, 5-793.5e, 5-793.5f, 5-793.63, 5-793.6f, 5-793.6n, 5-793.af, 5-793.be, 5-793.bh, 5-793.bn, 5-793.g9, 5-793.h1, 5-793.k1, 5-793.k3, 5-793.k4, 5-793.k6, 5-793.k7, 5-793.k9, 5-793.kf, 5-793.kh, 5-793.kk, 5-793.kn, 5-793.kr, 5-793.x1, 5-793.xp, 5-794.01, 5-794.03, 5-794.04, 5-794.06, 5-794.07, 5-794.09, 5-794.0e, 5-794.0f, 5-794.0h, 5-794.0j, 5-794.0k, 5-794.0n, 5-794.0p, 5-794.0r, 5-794.11, 5-794.13, 5-794.14, 5-794.16, 5-794.17, 5-794.19, 5-794.1e, 5-794.1f, 5-794.1h, 5-794.1j, 5-794.1k, 5-794.1n, 5-794.21, 5-794.23, 5-794.24, 5-794.26, 5-794.27, 5-794.29, 5-794.2f, 5-794.2h, 5-794.2j, 5-794.2k, 5-794.2n, 5-794.2p, 5-794.2r, 5-794.31, 5-794.33, 5-794.3h, 5-794.3k, 5-794.3n, 5-794.4e, 5-794.4f, 5-794.53, 5-794.56, 5-794.57, 5-794.5h, 5-794.5n, 5-794.5r, 5-794.7n, 5-794.ae, 5-794.af, 5-794.ah, 5-794.an, 5-794.be, 5-794.bf, 5-794.bh, 5-794.bn, 5-794.g1, 5-794.g3, 5-794.g4, 5-794.gr, 5-794.hn, 5-794.k1, 5-794.k3, 5-794.k4, 5-794.k6, 5-794.k7, 5-794.k9, 5-794.ke, 5-794.kf, 5-794.kh, 5-794.kj, 5-794.kk, 5-794.kn, 5-794.kr, 5-794.mh, 5-794.n3, 5-794.nk, 5-794.x1, 5-794.x3, 5-794.x4, 5-794.x7, 5-794.xj, 5-794.xk, 5-794.xn, 5-795.10, 5-795.1a, 5-795.1b, 5-795.1u, 5-795.1v, 5-795.1w, 5-795.1z, 5-795.20, 5-795.2b, 5-795.2c, 5-795.2u, 5-795.2v, 5-795.2w, 5-795.30, 5-795.3b, 5-795.3u, 5-795.3v, 5-795.g0, 5-795.hb, 5-795.k0, 5-795.ku, 5-795.kv, 5-795.kx, 5-795.xa, 5-796.00, 5-796.0a, 5-796.0b, 5-796.0c, 5-796.0u, 5-796.0v, 5-796.0z, 5-796.10, 5-796.1a, 5-796.1b, 5-796.1c, 5-796.1u, 5-796.1v, 5-796.1w, 5-796.20, 5-796.2b, 5-796.2c, 5-796.2z, 5-796.3b, 5-796.g0, 5-796.gv, 5-796.k0, 5-796.kb, 5-796.ku, 5-796.kv, 5-796.kx, 5-796.kz, 5-796.x0, 5-796.xc, 5-797.1s, 5-797.1t, 5-797.2s, 5-797.2t, 5-797.3t, 5-797.4s, 5-797.kt, 5-798.1, 5-798.2, 5-798.3, 5-798.4, 5-798.5, 5-798.6, 5-798.7, 5-799.1, 5-799.2, 5-799.3, 5-799.4, 5-799.x, 5-79a.0a, 5-79a.0e, 5-79a.0f, 5-79a.0g, 5-79a.0k, 5-79a.0p, 5-79a.15, 5-79a.16, 5-79a.17, 5-79a.1a, 5-79a.1b, 5-79a.1k, 5-79a.1m, 5-79a.1n, 5-79a.1p, 5-79a.1q, 5-79a.1r, 5-79a.64, 5-79a.67, 5-79a.68, 5-79a.6a, 5-79a.6f, 5-79a.6g, 5-79a.6h, 5-79a.6j, 5-79a.6k, 5-79a.6p, 5-79a.6s, 5-79a.g6, 5-79b.00, 5-79b.04, 5-79b.0e, 5-79b.0f, 5-79b.0g, 5-79b.0k, 5-79b.0n, 5-79b.0p, 5-79b.14, 5-79b.16, 5-79b.17, 5-79b.1a, 5-79b.1d, 5-79b.1f, 5-79b.1k, 5-79b.1m, 5-79b.1n, 5-79b.1p, 5-79b.1q, 5-79b.21, 5-79b.23, 5-79b.24, 5-79b.2f, 5-79b.2g, 5-79b.2p, 5-79b.64, 5-79b.67, 5-79b.6h, 5-79b.6k, 5-79b.6m, 5-79b.6s, 5-79b.6t, 5-79b.6x, 5-79b.g6, 5-79b.h0, 5-79b.h1, 5-79b.h4, 5-79b.hc, 5-79b.hd, 5-79b.hg, 5-79b.hp, 5-79b.hs, 5-79b.ht, 5-79b.xg, 5-79b.xp, 5-79c.0, 5-800.00, 5-800.05, 5-800.07, 5-800.0g, 5-800.0h, 5-800.0j, 5-800.0n, 5-800.0q, 5-800.0r, 5-800.0t, 5-800.10, 5-800.1g, 5-800.1h, 5-800.1k, 5-800.20, 5-800.24, 5-800.2f, 5-800.2g, 5-800.2h, 5-800.2q, 5-800.2s, 5-800.30, 5-800.31, 5-800.34, 5-800.3f, 5-800.3g, 5-800.3h, 5-800.3k, 5-800.3m, 5-800.3q, 5-800.3r, 5-800.3t, 5-800.40, 5-800.44, 5-800.47, 5-800.4h, 5-800.4m, 5-800.4r, 5-800.5g, 5-800.5h, 5-800.64, 5-800.6g, 5-800.70, 5-800.80, 5-800.84, 5-800.87, 5-800.8g, 5-800.8h, 5-800.8k, 5-800.8s, 5-800.8x, 5-800.a0, 5-800.af, 5-800.ag, 5-800.ah, 5-800.cg, 5-800.xg, 5-801.04, 5-801.0f, 5-801.0g, 5-801.0h, 5-801.30, 5-801.34, 5-801.36, 5-801.3h, 5-801.3j, 5-801.3k, 5-801.3n, 5-801.44, 5-801.46, 5-801.4g, 5-801.5, 5-801.6, 5-801.7, 5-801.e, 5-801.gh, 5-801.hh, 5-801.hk, 5-801.hq, 5-801.n3, 5-802.0, 5-802.1, 5-802.2, 5-802.4, 5-802.6, 5-802.7, 5-802.8, 5-802.9, 5-803.6, 5-803.7, 5-803.8, 5-803.a, 5-804.1, 5-804.5, 5-804.6, 5-804.7, 5-804.8, 5-805.0, 5-805.4, 5-805.7, 5-805.x, 5-806.3, 5-806.8, 5-806.9, 5-806.c, 5-806.d, 5-807.0, 5-807.1, 5-807.2, 5-807.4, 5-807.70, 5-807.71, 5-807.72, 5-807.73, 5-807.x, 5-808.0, 5-808.3, 5-808.6, 5-808.70, 5-808.71, 5-808.80, 5-808.a4, 5-808.a5, 5-808.a6, 5-808.a7, 5-808.a8, 5-809.26, 5-809.2k, 5-809.2m, 5-809.2p, 5-810.00, 5-810.0g, 5-810.0h, 5-810.10, 5-810.11, 5-810.1g, 5-810.1h, 5-810.1k, 5-810.20, 5-810.24, 5-810.2g, 5-810.2h, 5-810.2s, 5-810.40, 5-810.4g, 5-810.4h, 5-810.4k, 5-810.60, 5-810.6h, 5-810.6j, 5-810.7f, 5-810.7h, 5-810.9g, 5-810.9h, 5-810.9k, 5-811.0h, 5-811.1h, 5-811.20, 5-811.21, 5-811.2g, 5-811.2h, 5-811.2k, 5-811.3h, 5-812.00, 5-812.0g, 5-812.0h, 5-812.5, 5-812.6, 5-812.7, 5-812.eh, 5-812.fh, 5-812.kg, 5-812.kh, 5-812.xh, 5-813.0, 5-813.2, 5-813.7, 5-813.k, 5-814.0, 5-814.1, 5-814.2, 5-814.3, 5-814.5, 5-814.7, 5-814.9, 5-814.b, 5-815.1, 5-816.1, 5-819.1h, 5-819.x0, 5-820.00, 5-820.01, 5-820.02, 5-820.21, 5-820.22, 5-820.30, 5-820.40, 5-820.41, 5-820.50, 5-820.x2, 5-821.0, 5-821.10, 5-821.11, 5-821.13, 5-821.15, 5-821.18, 5-821.1x, 5-821.20, 5-821.24, 5-821.25, 5-821.26, 5-821.27, 5-821.29, 5-821.2a, 5-821.2b, 5-821.31, 5-821.33, 5-821.40, 5-821.42, 5-821.43, 5-821.52, 5-821.61, 5-821.63, 5-821.7, 5-821.9, 5-821.b, 5-821.c, 5-821.e, 5-821.f4, 5-821.fx, 5-821.g2, 5-822.f0, 5-822.g1, 5-822.h1, 5-823.0, 5-823.1b, 5-823.26, 5-823.27, 5-823.43, 5-823.7, 5-823.9, 5-823.a, 5-823.b0, 5-823.f0, 5-823.j, 5-823.k6, 5-823.m, 5-824.01, 5-824.20, 5-824.21, 5-824.3, 5-824.40, 5-825.00, 5-825.10, 5-825.12, 5-825.4, 5-825.k0, 5-825.k1, 5-825.kx, 5-825.x, 5-828.0, 5-828.40, 5-828.5, 5-829.1, 5-829.2, 5-829.5, 5-829.9, 5-829.c, 5-829.e, 5-829.f, 5-829.g, 5-829.h, 5-829.k0, 5-829.k1, 5-829.k2, 5-829.k3, 5-829.k4, 5-829.n, 5-830.0, 5-830.1, 5-830.2, 5-830.3, 5-830.4, 5-830.6, 5-830.7, 5-831.0, 5-831.2, 5-831.3, 5-831.4, 5-831.6, 5-831.7, 5-831.9, 5-832.0, 5-832.1, 5-832.2, 5-832.4, 5-832.5, 5-832.6, 5-832.7, 5-832.8, 5-832.9, 5-832.x, 5-832.y, 5-835.9, 5-835.a0, 5-835.b0, 5-835.d0, 5-836.30, 5-836.31, 5-836.32, 5-836.33, 5-836.34, 5-836.35, 5-836.36, 5-836.37, 5-836.38, 5-836.40, 5-836.41, 5-836.42, 5-836.43, 5-836.50, 5-836.51, 5-836.53, 5-836.55, 5-836.56, 5-836.x, 5-837.00, 5-837.01, 5-837.2, 5-837.4, 5-837.6, 5-837.a0, 5-837.x, 5-838.92, 5-838.b4, 5-838.x, 5-839.0, 5-839.10, 5-839.11, 5-839.2, 5-839.5, 5-839.60, 5-839.61, 5-839.62, 5-839.63, 5-839.8, 5-839.90, 5-839.a0, 5-839.a1, 5-839.a2, 5-839.a3, 5-839.j1, 5-83a.00, 5-83b.20, 5-83b.21, 5-83b.22, 5-83b.25, 5-83b.30, 5-83b.31, 5-83b.32, 5-83b.33, 5-83b.34, 5-83b.40, 5-83b.41, 5-83b.42, 5-83b.43, 5-83b.44, 5-83b.50, 5-83b.51, 5-83b.52, 5-83b.53, 5-83b.54, 5-83b.55, 5-83b.56, 5-83b.57, 5-83b.58, 5-83b.70, 5-83b.71, 5-83b.72, 5-83b.81, 5-83b.84, 5-83b.85, 5-83b.x3, 5-83b.x5, 5-83w.0, 5-83w.1, 5-83w.20, 5-840.07, 5-840.30, 5-840.33, 5-840.34, 5-840.35, 5-840.36, 5-840.40, 5-840.41, 5-840.44, 5-840.45, 5-840.47, 5-840.56, 5-840.60, 5-840.61, 5-840.62, 5-840.63, 5-840.64, 5-840.65, 5-840.66, 5-840.74, 5-840.80, 5-840.81, 5-840.82, 5-840.83, 5-840.84, 5-840.85, 5-840.94, 5-840.c4, 5-840.k4, 5-840.m0, 5-840.m3, 5-840.n4, 5-840.s0, 5-840.s1, 5-840.s3, 5-840.s4, 5-840.s6, 5-840.s7, 5-840.y, 5-841.00, 5-841.11, 5-841.14, 5-841.15, 5-841.41, 5-841.42, 5-841.44, 5-841.45, 5-841.46, 5-841.a2, 5-841.a6, 5-842.0, 5-842.40, 5-842.70, 5-842.91, 5-842.x, 5-843.6, 5-843.c, 5-844.22, 5-844.23, 5-844.30, 5-844.54, 5-844.57, 5-846.2, 5-849.1, 5-850.03, 5-850.06, 5-850.08, 5-850.09, 5-850.0a, 5-850.12, 5-850.13, 5-850.17, 5-850.18, 5-850.28, 5-850.3a, 5-850.49, 5-850.62, 5-850.63, 5-850.65, 5-850.66, 5-850.68, 5-850.69, 5-850.6a, 5-850.6x, 5-850.80, 5-850.82, 5-850.85, 5-850.86, 5-850.87, 5-850.88, 5-850.89, 5-850.8a, 5-850.a0, 5-850.a2, 5-850.a3, 5-850.a5, 5-850.a8, 5-850.b2, 5-850.b3, 5-850.b5, 5-850.b6, 5-850.b7, 5-850.b8, 5-850.b9, 5-850.ba, 5-850.bx, 5-850.c2, 5-850.c3, 5-850.c8, 5-850.c9, 5-850.ca, 5-850.cx, 5-850.d0, 5-850.d2, 5-850.d3, 5-850.d5, 5-850.d6, 5-850.d7, 5-850.d8, 5-850.d9, 5-850.da, 5-850.dx, 5-850.e0, 5-850.e2, 5-850.e3, 5-850.e9, 5-850.ea, 5-850.ex, 5-850.f3, 5-850.f9, 5-850.fa, 5-850.fx, 5-850.g0, 5-850.g3, 5-850.g9, 5-850.h8, 5-850.h9, 5-850.j8, 5-851.09, 5-851.11, 5-851.12, 5-851.52, 5-851.53, 5-851.58, 5-851.59, 5-851.5a, 5-851.68, 5-851.69, 5-851.6a, 5-851.7a, 5-851.82, 5-851.83, 5-851.85, 5-851.88, 5-851.89, 5-851.93, 5-851.97, 5-851.98, 5-851.99, 5-851.9a, 5-851.9x, 5-851.aa, 5-851.b3, 5-851.b9, 5-851.ba, 5-851.c2, 5-851.c3, 5-851.c8, 5-851.c9, 5-851.ca, 5-851.cx, 5-852.01, 5-852.03, 5-852.09, 5-852.0a, 5-852.13, 5-852.19, 5-852.1x, 5-852.43, 5-852.47, 5-852.48, 5-852.49, 5-852.53, 5-852.61, 5-852.63, 5-852.65, 5-852.67, 5-852.68, 5-852.69, 5-852.6a, 5-852.78, 5-852.79, 5-852.87, 5-852.88, 5-852.89, 5-852.8a, 5-852.93, 5-852.97, 5-852.9a, 5-852.a2, 5-852.a3, 5-852.a8, 5-852.a9, 5-852.aa, 5-852.c9, 5-852.f8, 5-852.g0, 5-853.00, 5-853.02, 5-853.03, 5-853.05, 5-853.08, 5-853.10, 5-853.11, 5-853.12, 5-853.13, 5-853.15, 5-853.17, 5-853.18, 5-853.19, 5-853.1a, 5-853.1x, 5-853.53, 5-853.73, 5-853.x2, 5-853.x3, 5-853.x5, 5-853.x6, 5-853.x8, 5-853.x9, 5-853.xa, 5-854.32, 5-854.38, 5-854.42, 5-854.58, 5-855.01, 5-855.02, 5-855.03, 5-855.07, 5-855.08, 5-855.09, 5-855.0a, 5-855.11, 5-855.12, 5-855.13, 5-855.18, 5-855.19, 5-855.1a, 5-855.1x, 5-855.21, 5-855.53, 5-855.59, 5-855.63, 5-855.71, 5-855.7a, 5-855.81, 5-855.9a, 5-855.b2, 5-855.b9, 5-855.ba, 5-856.00, 5-856.01, 5-856.02, 5-856.03, 5-856.05, 5-856.06, 5-856.08, 5-856.09, 5-856.28, 5-856.2a, 5-856.a6, 5-857.04, 5-857.17, 5-857.50, 5-857.76, 5-857.77, 5-857.85, 5-857.88, 5-857.89, 5-857.8a, 5-857.98, 5-857.99, 5-858.01, 5-858.08, 5-858.25, 5-858.26, 5-858.30, 5-858.35, 5-858.39, 5-858.53, 5-858.54, 5-858.59, 5-858.5a, 5-858.73, 5-858.74, 5-858.78, 5-858.79, 5-858.7a, 5-858.89, 5-858.8a, 5-859.11, 5-859.12, 5-859.13, 5-859.18, 5-859.19, 5-859.1a, 5-859.x2, 5-859.x9, 5-859.xa, 5-860.3, 5-862.2, 5-862.4, 5-863.1, 5-863.3, 5-863.5, 5-863.6, 5-864.1, 5-864.2, 5-864.4, 5-864.5, 5-864.7, 5-864.9, 5-864.a, 5-865.4, 5-865.7, 5-865.8, 5-866.3, 5-866.4, 5-866.5, 5-869.1, 5-869.2, 5-86a.00, 5-86a.01, 5-86a.02, 5-86a.03, 5-86a.10, 5-86a.11, 5-86a.12, 5-86a.13, 5-86a.20, 5-86a.21, 5-886.0, 5-890.27, 5-890.28, 5-890.29, 5-890.2f, 5-892.04, 5-892.06, 5-892.07, 5-892.08, 5-892.09, 5-892.0a, 5-892.0b, 5-892.0c, 5-892.0d, 5-892.0e, 5-892.0f, 5-892.0g, 5-892.0x, 5-892.10, 5-892.14, 5-892.15, 5-892.16, 5-892.17, 5-892.18, 5-892.19, 5-892.1a, 5-892.1b, 5-892.1c, 5-892.1d, 5-892.1e, 5-892.1f, 5-892.1g, 5-892.1x, 5-892.20, 5-892.24, 5-892.25, 5-892.27, 5-892.28, 5-892.29, 5-892.2a, 5-892.2b, 5-892.2d, 5-892.2e, 5-892.2f, 5-892.2g, 5-892.36, 5-892.3a, 5-892.3b, 5-892.3d, 5-892.3e, 5-892.3f, 5-892.3g, 5-892.47, 5-892.4b, 5-892.4e, 5-892.4f, 5-892.xa, 5-894.04, 5-894.06, 5-894.08, 5-894.0b, 5-894.0e, 5-894.0f, 5-894.0g, 5-894.14, 5-894.17, 5-894.18, 5-894.19, 5-894.1a, 5-894.1c, 5-894.1g, 5-894.34, 5-895.07, 5-895.08, 5-895.09, 5-895.0a, 5-895.0f, 5-895.0g, 5-895.14, 5-895.24, 5-895.25, 5-895.28, 5-895.2e, 5-895.2f, 5-895.38, 5-895.44, 5-896.00, 5-896.04, 5-896.05, 5-896.06, 5-896.07, 5-896.08, 5-896.09, 5-896.0a, 5-896.0b, 5-896.0c, 5-896.0d, 5-896.0e, 5-896.0f, 5-896.0g, 5-896.10, 5-896.14, 5-896.15, 5-896.16, 5-896.17, 5-896.18, 5-896.19, 5-896.1a, 5-896.1b, 5-896.1c, 5-896.1d, 5-896.1e, 5-896.1f, 5-896.1g, 5-896.1x, 5-896.25, 5-896.26, 5-896.27, 5-896.28, 5-896.2a, 5-896.2b, 5-896.2d, 5-896.2e, 5-896.2f, 5-896.2g, 5-896.x8, 5-896.xe, 5-896.xf, 5-896.xg, 5-896.xx, 5-896.y, 5-897.0, 5-898.2, 5-898.3, 5-898.4, 5-898.5, 5-898.6, 5-898.8, 5-898.9, 5-898.a, 5-898.x, 5-900.00, 5-900.04, 5-900.05, 5-900.06, 5-900.07, 5-900.08, 5-900.09, 5-900.0a, 5-900.0b, 5-900.0c, 5-900.0d, 5-900.0e, 5-900.0f, 5-900.0g, 5-900.0x, 5-900.10, 5-900.14, 5-900.15, 5-900.16, 5-900.17, 5-900.18, 5-900.19, 5-900.1a, 5-900.1b, 5-900.1c, 5-900.1d, 5-900.1e, 5-900.1f, 5-900.1g, 5-900.1x, 5-900.x0, 5-900.x4, 5-900.x7, 5-900.y, 5-901.07, 5-901.0a, 5-901.0b, 5-901.0d, 5-901.0e, 5-901.0f, 5-902.08, 5-902.0f, 5-902.0g, 5-902.1e, 5-902.1f, 5-902.1g, 5-902.44, 5-902.47, 5-902.48, 5-902.49, 5-902.4a, 5-902.4b, 5-902.4c, 5-902.4d, 5-902.4e, 5-902.4f, 5-902.4g, 5-902.58, 5-902.59, 5-902.5b, 5-902.5e, 5-902.5f, 5-902.5g, 5-902.c6, 5-902.c7, 5-902.c8, 5-902.ca, 5-902.cb, 5-902.cc, 5-902.cd, 5-902.ce, 5-902.dg, 5-902.e8, 5-902.ee, 5-902.ef, 5-902.eg, 5-902.g9, 5-903.04, 5-903.14, 5-903.54, 5-903.5b, 5-903.5c, 5-903.5d, 5-903.64, 5-903.68, 5-903.6f, 5-903.8d, 5-904.0a, 5-904.0e, 5-904.17, 5-904.18, 5-905.0f, 5-905.0g, 5-905.17, 5-905.18, 5-905.19, 5-905.1d, 5-907.18, 5-907.58, 5-907.5f, 5-907.5g, 5-907.xf, 5-907.xg, 5-908.0, 5-908.1, 5-908.2, 5-909.3, 5-909.x5, 5-909.x8, 5-910, 5-911.2g, 5-916.0a, 5-916.2f, 5-916.78, 5-916.7e, 5-916.7f, 5-916.a0, 5-916.a1, 5-916.a2, 5-916.a4, 5-916.a5, 5-916.a6, 5-916.ax, 5-916.c8, 5-916.x6, 5-916.x8, 5-920.0f, 5-921.00, 5-921.01, 5-921.02, 5-921.03, 5-921.04, 5-921.05, 5-921.06, 5-921.07, 5-921.08, 5-921.09, 5-921.0a, 5-921.0b, 5-921.0c, 5-921.0d, 5-921.0e, 5-921.0f, 5-921.0h, 5-921.0j, 5-921.0k, 5-921.0m, 5-921.14, 5-921.17, 5-921.18, 5-921.19, 5-921.1e, 5-921.26, 5-921.27, 5-921.28, 5-921.29, 5-921.2a, 5-921.2b, 5-921.2c, 5-921.2d, 5-921.2e, 5-921.2f, 5-921.2g, 5-921.2k, 5-921.35, 5-921.36, 5-921.37, 5-921.38, 5-921.39, 5-921.3a, 5-921.3b, 5-921.3c, 5-921.3d, 5-921.3e, 5-921.3f, 5-921.3g, 5-921.3h, 5-921.3j, 5-921.x4, 5-921.x6, 5-921.x7, 5-921.x8, 5-921.x9, 5-921.xe, 5-921.xj, 5-922.0, 5-922.1, 5-923.09, 5-923.29, 5-923.66, 5-923.67, 5-923.68, 5-923.69, 5-923.6a, 5-923.6b, 5-923.6c, 5-923.6d, 5-923.6e, 5-923.6f, 5-923.6k, 5-923.79, 5-923.7c, 5-923.7k, 5-923.b0, 5-923.b1, 5-923.b2, 5-923.b4, 5-923.b5, 5-923.b6, 5-923.b7, 5-923.b8, 5-923.b9, 5-923.ba, 5-923.bj, 5-923.d7, 5-923.d8, 5-923.d9, 5-924.06, 5-924.07, 5-924.08, 5-924.0b, 5-924.0e, 5-924.0f, 5-924.0h, 5-924.0j, 5-925.05, 5-925.06, 5-925.07, 5-925.08, 5-925.09, 5-925.0a, 5-925.0b, 5-925.0c, 5-925.0d, 5-925.0e, 5-925.0f, 5-925.0g, 5-925.0j, 5-925.76, 5-925.77, 5-925.78, 5-925.7a, 5-925.7b, 5-925.7c, 5-925.a6, 5-925.a7, 5-925.a8, 5-925.a9, 5-925.ac, 5-925.ae, 5-925.af, 5-925.d9, 5-925.e5, 5-925.e7, 5-925.e8, 5-925.e9, 5-925.ee, 5-926.0h, 5-928.01, 5-928.02, 5-928.03, 5-928.05, 5-928.0e, 5-929.1e, 5-929.1f, 5-929.x7, 5-929.x8, 5-929.x9, 5-930.00, 5-930.01, 5-930.3, 5-930.4, 5-931.0, 5-931.1, 5-932.13, 5-932.16, 5-932.17, 5-934.0, 5-934.2, 5-935.x, 5-93a.0, 5-981, 5-982.0, 5-982.1, 5-982.2, 5-982.x, 5-982.y, 5-983, 5-984, 5-985.0, 5-985.1, 5-985.6, 5-986.0x, 5-986.x, 5-986.y, 5-987.x, 5-988.0, 5-988.1, 5-988.3, 5-989, 5-98c.0, 5-98c.1, 5-98c.2, 5-98c.70, 5-98c.71, 5-98e, 5-98k.2, 5-995, 6-001.92, 6-001.93, 6-001.97, 6-001.9p, 6-001.c2, 6-001.f9, 6-001.h4, 6-001.h5, 6-001.ha, 6-002.52, 6-002.54, 6-002.56, 6-002.57, 6-002.59, 6-002.72, 6-002.73, 6-002.9d, 6-002.b0, 6-002.e4, 6-002.j3, 6-002.ja, 6-002.p1, 6-002.p2, 6-002.p3, 6-002.p4, 6-002.p6, 6-002.p7, 6-002.p9, 6-002.pa, 6-002.pc, 6-002.pd, 6-002.pe, 6-002.pf, 6-002.pg, 6-002.pj, 6-002.pn, 6-002.pp, 6-002.qc, 6-002.qg, 6-002.qn, 6-002.r2, 6-002.r5, 6-002.r8, 6-002.r9, 6-002.rb, 6-002.rc, 6-002.rd, 6-002.re, 6-002.rf, 6-002.rh, 6-003.30, 6-003.8, 6-003.g0, 6-003.g1, 6-003.k2, 6-003.p3, 6-004.c, 6-004.d, 6-005.04, 6-005.m3, 6-005.m7, 6-005.n1, 6-006.21, 6-006.23, 6-006.25, 6-006.a0, 6-006.a2, 6-006.d, 6-006.j6, 6-006.jh, 6-007.02, 6-007.05, 6-007.62, 6-007.63, 6-007.64, 6-007.a, 6-007.k3, 6-007.p4, 6-007.p7, 6-008.f, 6-008.j, 6-008.m2, 6-008.m8, 6-009.3, 6-009.38, 6-009.4, 6-009.ae, 6-009.j, 6-009.p6, 6-009.rc, 6-00a.0, 6-00a.9, 6-00b.0, 6-00b.9, 6-00b.c, 6-00c.1, 6-00c.7, 6-00d.e, 6-00f.p1, 6-00f.p2, 6-00f.p5, 6-00f.p7, 8-017.0, 8-018.0, 8-018.1, 8-020.0, 8-020.5, 8-020.7, 8-020.8, 8-020.d, 8-020.x, 8-100.4, 8-100.40, 8-100.8, 8-101.1, 8-101.2, 8-101.3, 8-101.x, 8-102.1, 8-102.2, 8-102.5, 8-102.6, 8-123.0, 8-123.1, 8-123.x, 8-125.0, 8-125.1, 8-125.2, 8-128, 8-132.1, 8-132.2, 8-132.3, 8-132.x, 8-133.0, 8-137.00, 8-137.03, 8-137.10, 8-139.x, 8-144.0, 8-144.1, 8-144.2, 8-146.0, 8-146.1, 8-146.2, 8-148.0, 8-148.1, 8-148.3, 8-148.x, 8-151.1, 8-151.4, 8-152.0, 8-152.1, 8-152.2, 8-152.x, 8-153, 8-154.0, 8-154.1, 8-154.2, 8-154.3, 8-158.0, 8-158.4, 8-158.g, 8-158.h, 8-158.x, 8-159.2, 8-159.x, 8-171.0, 8-172, 8-173.10, 8-176.1, 8-176.2, 8-178.0, 8-178.4, 8-178.5, 8-178.g, 8-178.h, 8-178.k, 8-178.m, 8-178.r, 8-178.t, 8-179.0, 8-179.3, 8-179.x, 8-179.y, 8-190.20, 8-190.21, 8-190.22, 8-190.23, 8-190.30, 8-190.31, 8-190.32, 8-190.33, 8-190.40, 8-190.41, 8-190.42, 8-191.00, 8-191.01, 8-191.10, 8-191.11, 8-191.20, 8-191.21, 8-191.40, 8-191.70, 8-191.71, 8-191.76, 8-192.04, 8-192.09, 8-192.0c, 8-192.0e, 8-192.0f, 8-192.18, 8-192.19, 8-192.1b, 8-192.1e, 8-192.1f, 8-192.1g, 8-192.2a, 8-192.36, 8-192.37, 8-192.x8, 8-192.xf, 8-200.0, 8-200.1, 8-200.2, 8-200.3, 8-200.4, 8-200.5, 8-200.6, 8-200.8, 8-200.9, 8-200.c, 8-200.d, 8-200.e, 8-200.g, 8-200.m, 8-200.n, 8-200.r, 8-200.s, 8-200.t, 8-200.w, 8-201.0, 8-201.1, 8-201.2, 8-201.3, 8-201.4, 8-201.5, 8-201.6, 8-201.7, 8-201.8, 8-201.c, 8-201.d, 8-201.g, 8-201.h, 8-201.k, 8-201.m, 8-201.p, 8-201.q, 8-201.r, 8-201.t, 8-201.u, 8-201.x, 8-202.0, 8-202.2, 8-210, 8-310.1, 8-310.3, 8-310.4, 8-310.5, 8-310.x, 8-310.y, 8-390.0, 8-390.1, 8-390.4, 8-390.5, 8-390.6, 8-390.x, 8-400.2, 8-401.2, 8-401.4, 8-401.x, 8-401.y, 8-410.0, 8-500, 8-504, 8-506, 8-522.30, 8-522.31, 8-522.60, 8-522.90, 8-522.91, 8-522.b0, 8-522.b1, 8-523.00, 8-527.0, 8-527.1, 8-527.2, 8-527.6, 8-527.8, 8-529.3, 8-529.7, 8-541.0, 8-542.11, 8-542.12, 8-542.13, 8-542.33, 8-543.13, 8-543.32, 8-543.34, 8-543.42, 8-544.0, 8-544.1, 8-547.0, 8-547.30, 8-547.31, 8-548.0, 8-550.0, 8-550.1, 8-550.2, 8-559.40, 8-559.80, 8-560.1, 8-607.0, 8-640.0, 8-640.1, 8-641, 8-642, 8-643, 8-700.1, 8-701, 8-704, 8-706, 8-711.21, 8-712.0, 8-712.1, 8-713.0, 8-714.00, 8-714.01, 8-714.02, 8-716.01, 8-718.82, 8-718.83, 8-718.84, 8-718.85, 8-718.86, 8-721.0, 8-721.1, 8-721.2, 8-771, 8-772, 8-779, 8-800.0, 8-800.66, 8-800.c0, 8-800.c1, 8-800.c2, 8-800.c3, 8-800.c4, 8-800.c5, 8-800.c6, 8-800.c7, 8-800.c8, 8-800.c9, 8-800.cd, 8-800.ch, 8-800.f0, 8-800.f1, 8-800.f2, 8-800.f5, 8-800.g0, 8-800.g1, 8-800.g2, 8-800.g3, 8-800.g4, 8-800.g5, 8-800.g6, 8-800.g7, 8-800.g8, 8-800.g9, 8-800.ga, 8-800.gb, 8-803.2, 8-810.6c, 8-810.78, 8-810.7g, 8-810.89, 8-810.8c, 8-810.97, 8-810.9a, 8-810.9b, 8-810.c5, 8-810.e8, 8-810.e9, 8-810.ea, 8-810.j, 8-810.j3, 8-810.j4, 8-810.j5, 8-810.j6, 8-810.j7, 8-810.j8, 8-810.j9, 8-810.ja, 8-810.jb, 8-810.jc, 8-810.jd, 8-810.je, 8-810.jh, 8-810.jk, 8-810.w3, 8-810.wb, 8-810.wc, 8-810.wd, 8-810.wf, 8-810.wn, 8-812.50, 8-812.51, 8-812.52, 8-812.53, 8-812.54, 8-812.55, 8-812.56, 8-812.57, 8-812.58, 8-812.59, 8-812.5a, 8-812.5b, 8-812.5d, 8-812.60, 8-812.61, 8-812.62, 8-812.63, 8-812.64, 8-812.80, 8-812.81, 8-820.01, 8-820.04, 8-821.2, 8-822, 8-824, 8-826.02, 8-831.0, 8-831.00, 8-831.01, 8-831.04, 8-831.2, 8-831.20, 8-831.21, 8-831.23, 8-831.24, 8-831.5, 8-831.y, 8-832.0, 8-835.20, 8-835.30, 8-835.32, 8-835.33, 8-835.8, 8-835.h, 8-835.j, 8-836.00, 8-836.08, 8-836.0c, 8-836.0e, 8-836.0m, 8-836.0s, 8-836.6j, 8-836.7c, 8-836.7k, 8-836.80, 8-836.8k, 8-836.90, 8-836.9h, 8-836.kd, 8-836.kh, 8-836.kk, 8-836.m0, 8-836.ma, 8-836.mh, 8-836.mk, 8-836.n1, 8-836.n2, 8-836.n3, 8-836.n4, 8-836.n6, 8-836.n7, 8-836.n8, 8-836.n9, 8-836.ne, 8-836.nj, 8-836.nm, 8-836.nq, 8-836.px, 8-837.00, 8-837.01, 8-837.a0, 8-837.k3, 8-837.m0, 8-837.m1, 8-837.m5, 8-837.m8, 8-837.ma, 8-837.s0, 8-837.t, 8-838.60, 8-839.10, 8-839.46, 8-839.48, 8-839.a5, 8-839.x, 8-83a.30, 8-83a.35, 8-83a.36, 8-83b.00, 8-83b.07, 8-83b.0b, 8-83b.13, 8-83b.22, 8-83b.2x, 8-83b.31, 8-83b.33, 8-83b.34, 8-83b.35, 8-83b.36, 8-83b.3x, 8-83b.50, 8-83b.70, 8-83b.71, 8-83b.72, 8-83b.80, 8-83b.82, 8-83b.84, 8-83b.85, 8-83b.86, 8-83b.88, 8-83b.ba, 8-83b.bb, 8-83b.bx, 8-83b.c2, 8-83b.c6, 8-83b.d, 8-83b.e1, 8-83b.j, 8-83b.s, 8-83b.x, 8-83c.61, 8-83c.70, 8-83c.8, 8-840.0c, 8-840.0k, 8-840.0q, 8-840.0s, 8-840.18, 8-841.0s, 8-842.02, 8-842.08, 8-844.0x, 8-84b.00, 8-851.50, 8-852.00, 8-852.01, 8-852.03, 8-852.05, 8-852.0b, 8-852.30, 8-852.31, 8-852.35, 8-852.4, 8-853.70, 8-853.71, 8-853.74, 8-853.76, 8-853.80, 8-853.81, 8-853.82, 8-853.83, 8-853.84, 8-854.2, 8-854.3, 8-854.4, 8-854.5, 8-854.60, 8-854.61, 8-854.62, 8-854.63, 8-854.64, 8-854.66, 8-854.67, 8-854.70, 8-854.71, 8-854.72, 8-854.74, 8-855.3, 8-855.4, 8-857.16, 8-857.17, 8-858, 8-85b, 8-900, 8-902, 8-903, 8-910, 8-914.00, 8-914.02, 8-914.0x, 8-914.10, 8-914.11, 8-914.12, 8-915, 8-917.03, 8-917.0x, 8-917.13, 8-917.1x, 8-919, 8-91b, 8-920, 8-923.1, 8-924, 8-925.00, 8-925.01, 8-925.21, 8-925.23, 8-930, 8-931.0, 8-932, 8-933, 8-971.0, 8-971.1, 8-97d.1, 8-980.0, 8-980.10, 8-980.11, 8-980.20, 8-980.21, 8-980.30, 8-980.31, 8-980.40, 8-980.41, 8-980.50, 8-980.60, 8-980.7, 8-980.9, 8-981.0, 8-981.1, 8-981.30, 8-981.31, 8-981.32, 8-981.33, 8-984.0, 8-987.00, 8-987.01, 8-987.02, 8-987.03, 8-987.10, 8-987.11, 8-987.12, 8-987.13, 8-989.0, 8-989.1, 8-989.2, 8-989.3, 8-989.5, 8-989.6, 8-98d.0, 8-98d.1, 8-98d.3, 8-98d.4, 8-98d.9, 8-98f.0, 8-98f.10, 8-98f.11, 8-98f.20, 8-98f.21, 8-98f.30, 8-98f.31, 8-98f.40, 8-98f.41, 8-98f.50, 8-98f.51, 8-98f.60, 8-98f.61, 8-98f.7, 8-98f.8, 8-98f.9, 8-98f.a, 8-98g.00, 8-98g.01, 8-98g.02, 8-98g.03, 8-98g.04, 8-98g.10, 8-98g.11, 8-98g.12, 8-98g.13, 8-98g.14, 8-98h.00, 8-98h.01, 8-98h.02, 8-98h.03, 8-98h.04, 8-98h.05, 8-98h.06, 8-98h.07, 8-98h.0a, 9-200.00, 9-200.01, 9-200.02, 9-200.1, 9-200.5, 9-200.6, 9-200.7, 9-200.8, 9-200.9, 9-200.b, 9-261, 9-310, 9-311, 9-320, 9-500.0, 9-607, 9-649.0, 9-984.6, 9-984.7, 9-984.8, 9-984.9, 9-984.a, 9-984.b, 9-990, 9-999.01, 9-999.04, 9-999.08, ICG, PCR, POC

**ICD:**

A01.0, A02.0, A02.1, A02.2, A04.5, A04.70, A04.71, A04.72, A04.73, A04.79, A04.9, A06.0, A08.0, A08.1, A08.3, A09.0, A09.9, A16.0, A16.2, A17.0, A18.0, A19.2, A23.0, A26.0, A28.1, A31.88, A38, A40.0, A40.2, A40.8, A41.0, A41.1, A41.4, A41.51, A41.52, A41.58, A41.8, A41.9, A46, A48.1, A48.8, A49.0, A49.1, A49.8, A49.9, A53.0, A60.0, A69.2, A81.2, B00.1, B00.2, B00.4, B00.78, B00.8, B01.9, B02.0, B02.2, B02.3, B02.8, B02.9, B07, B08.4, B08.8, B09, B15.9, B16.9, B17.1, B18.1, B18.11, B18.13, B18.19, B18.2, B18.9, B20, B24, B25.88, B25.9, B27.0, B33.3, B34.2, B34.88, B34.9, B35.1, B35.3, B35.4, B35.6, B36.0, B36.8, B37.0, B37.1, B37.2, B37.3, B37.4, B37.6, B37.7, B37.81, B37.88, B37.9, B44.0, B44.1, B44.9, B46.5, B48.7, B48.80, B49, B58.0, B76.9, B80, B85.0, B85.4, B86, B87.0, B87.1, B87.3, B87.9, B88.2, B88.8, B91, B95.0, B95.1, B95.2, B95.3, B95.41, B95.42, B95.48, B95.5, B95.6, B95.7, B95.8, B95.90, B95.91, B96.0, B96.2, B96.3, B96.5, B96.6, B96.7, B96.8, B97.2, B97.4, B97.8, B98.0, B99, C00.1, C00.6, C01, C02.9, C04.0, C07, C08.8, C10.3, C10.8, C10.9, C13.8, C13.9, C15.2, C15.3, C15.4, C15.5, C15.8, C16.0, C16.2, C16.3, C16.9, C17.2, C18.0, C18.2, C18.7, C18.9, C20, C21.1, C22.0, C22.1, C22.7, C22.9, C23, C24.0, C24.1, C25.0, C25.1, C25.2, C25.9, C31.0, C32.0, C32.8, C32.9, C34.0, C34.1, C34.2, C34.3, C34.8, C34.9, C38.4, C40.0, C40.2, C40.9, C41.1, C41.2, C41.8, C41.9, C43.2, C43.6, C43.8, C43.9, C44.2, C44.3, C44.4, C44.7, C44.9, C47.8, C48.0, C49.1, C49.2, C49.4, C49.5, C49.6, C49.9, C50.0, C50.1, C50.3, C50.4, C50.5, C50.8, C50.9, C52, C53.0, C53.9, C54.1, C56, C60.9, C61, C62.1, C62.9, C64, C66, C67.2, C67.8, C67.9, C68.0, C71.1, C71.2, C73, C74.0, C74.1, C74.9, C77.0, C77.1, C77.2, C77.3, C77.5, C77.8, C78.0, C78.1, C78.2, C78.4, C78.6, C78.7, C79.0, C79.2, C79.3, C79.4, C79.5, C79.7, C79.81, C79.88, C80.0, C80.9, C81.1, C81.2, C81.7, C81.9, C82.2, C82.3, C82.7, C83.0, C83.1, C83.3, C83.7, C84.4, C85.1, C85.7, C88.40, C90.00, C91.00, C91.10, C91.11, C91.81, C92.00, C92.50, C92.80, C93.10, C96.6, C97, D01.9, D04.4, D05.1, D06.0, D10.7, D12.0, D12.2, D12.3, D12.8, D13.0, D13.1, D16.0, D16.1, D16.2, D16.3, D16.8, D16.9, D17.1, D17.3, D17.7, D18.02, D18.03, D18.05, D18.08, D21.2, D21.9, D22.4, D22.6, D22.7, D25.1, D25.9, D29.1, D30.3, D31.4, D32.0, D32.1, D32.9, D33.3, D35.2, D35.4, D36.7, D37.0, D37.1, D37.6, D37.70, D37.78, D38.1, D38.3, D39.1, D39.7, D40.0, D41.0, D41.4, D42.0, D42.1, D43.0, D43.1, D43.4, D44.0, D44.1, D45, D46.1, D46.4, D46.7, D46.9, D47.0, D47.1, D47.2, D47.3, D47.4, D47.9, D48.0, D48.1, D48.2, D48.5, D48.6, D48.7, D48.9, D50.0, D50.8, D50.9, D51.0, D51.3, D51.8, D51.9, D52.0, D52.1, D52.8, D52.9, D53.0, D53.1, D53.8, D53.9, D56.0, D56.3, D57.0, D57.1, D61.10, D61.19, D61.9, D62, D63.0, D63.8, D64.8, D64.9, D65.0, D65.1, D65.2, D66, D67, D68.00, D68.01, D68.20, D68.21, D68.22, D68.23, D68.25, D68.31, D68.33, D68.34, D68.35, D68.38, D68.4, D68.5, D68.6, D68.8, D68.9, D69.3, D69.41, D69.52, D69.53, D69.57, D69.58, D69.59, D69.60, D69.61, D69.80, D69.9, D70.10, D70.11, D70.13, D70.14, D70.18, D70.19, D70.3, D70.6, D70.7, D72.1, D72.8, D73.0, D73.5, D74.8, D75.8, D75.9, D80.9, D81.1, D86.0, D86.2, D86.3, D86.8, D90, E01.1, E03.1, E03.2, E03.8, E03.9, E04.0, E04.1, E04.2, E04.8, E04.9, E05.0, E05.1, E05.2, E05.4, E05.5, E05.8, E05.9, E06.1, E06.3, E10.01, E10.11, E10.20, E10.40, E10.60, E10.61, E10.72, E10.73, E10.74, E10.75, E10.90, E10.91, E11.01, E11.11, E11.20, E11.21, E11.30, E11.31, E11.40, E11.41, E11.50, E11.51, E11.60, E11.61, E11.72, E11.73, E11.74, E11.75, E11.90, E11.91, E12.60, E13.20, E13.41, E13.61, E13.90, E13.91, E14.60, E14.74, E14.90, E14.91, E16.0, E16.1, E16.2, E20.8, E21.0, E21.1, E21.3, E22.2, E23.0, E23.2, E23.6, E24.8, E27.1, E27.3, E27.4, E28.2, E34.4, E41, E43, E44.0, E44.1, E45, E46, E51.2, E51.8, E53.1, E53.8, E53.9, E55.0, E55.9, E58, E60, E61.1, E61.2, E63.1, E63.8, E63.9, E64.0, E64.9, E66.00, E66.01, E66.02, E66.04, E66.05, E66.06, E66.07, E66.08, E66.09, E66.16, E66.18, E66.22, E66.28, E66.80, E66.81, E66.82, E66.84, E66.86, E66.87, E66.89, E66.90, E66.91, E66.92, E66.94, E66.95, E66.96, E70.2, E72.0, E72.2, E73.8, E73.9, E74.0, E76.0, E78.0, E78.1, E78.2, E78.4, E78.5, E78.9, E79.0, E83.1, E83.30, E83.38, E83.39, E83.4, E83.58, E84.87, E84.9, E85.0, E85.4, E85.9, E86, E87.0, E87.1, E87.2, E87.3, E87.5, E87.6, E87.7, E88.0, E88.1, E88.29, E88.3, E88.8, E89.0, E89.2, E89.3, E89.6, F00.0, F00.1, F00.2, F00.9, F01.1, F01.2, F01.3, F01.8, F01.9, F02.0, F02.2, F02.3, F02.8, F03, F05.0, F05.1, F05.8, F05.9, F06.0, F06.2, F06.3, F06.7, F06.8, F06.9, F07.0, F07.2, F07.8, F10.0, F10.1, F10.2, F10.3, F10.4, F10.5, F10.6, F11.0, F11.1, F11.2, F11.3, F11.4, F11.5, F12.0, F12.1, F12.2, F12.3, F12.5, F13.0, F13.1, F13.2, F13.3, F13.4, F13.9, F14.0, F14.1, F14.2, F14.3, F14.4, F15.0, F15.1, F15.2, F15.3, F15.4, F15.5, F16.1, F17.1, F17.2, F17.3, F19.0, F19.1, F19.2, F19.3, F19.4, F19.5, F19.9, F20.0, F20.1, F20.3, F20.5, F20.8, F20.9, F22.0, F22.8, F22.9, F23.0, F23.1, F23.2, F23.3, F23.8, F23.9, F25.0, F25.1, F25.2, F25.8, F25.9, F28, F29, F31.1, F31.2, F31.3, F31.4, F31.6, F31.8, F31.9, F32.0, F32.1, F32.2, F32.3, F32.8, F32.9, F33.0, F33.1, F33.2, F33.3, F33.4, F33.8, F33.9, F34.1, F38.8, F40.00, F40.1, F40.2, F40.8, F41.0, F41.1, F41.2, F41.8, F41.9, F42.0, F42.2, F42.8, F42.9, F43.0, F43.1, F43.2, F43.9, F44.2, F44.4, F44.5, F44.6, F44.81, F44.88, F45.0, F45.1, F45.33, F45.40, F45.41, F45.8, F45.9, F50.1, F50.9, F51.1, F51.2, F51.3, F51.5, F54, F55.5, F55.9, F60.0, F60.1, F60.30, F60.31, F60.8, F61, F63.8, F70.0, F70.1, F70.9, F71.1, F72.9, F74.1, F79.0, F79.8, F79.9, F80.1, F80.8, F80.9, F81.2, F81.3, F82.0, F82.1, F83, F84.0, F84.2, F84.5, F84.8, F84.9, F89, F90.0, F90.1, F90.9, F91.1, F91.2, F91.3, F92.0, F95.1, F95.2, F98.08, F98.2, F98.5, F98.80, F99, G00.2, G00.8, G01, G03.8, G03.9, G04.8, G04.9, G05.1, G06.0, G06.1, G06.2, G08, G09, G10, G11.1, G12.1, G12.2, G13.8, G20.00, G20.01, G20.10, G20.11, G20.21, G20.90, G20.91, G21.1, G21.4, G21.8, G21.9, G23.0, G23.1, G23.2, G23.3, G23.8, G23.9, G24.0, G24.3, G24.8, G24.9, G25.0, G25.2, G25.3, G25.5, G25.81, G25.9, G30.0, G30.1, G30.8, G30.9, G31.0, G31.1, G31.2, G31.82, G32.0, G32.8, G35.0, G35.10, G35.11, G35.20, G35.21, G35.30, G35.31, G35.9, G37.8, G40.09, G40.1, G40.2, G40.3, G40.4, G40.5, G40.6, G40.7, G40.8, G40.9, G41.0, G41.2, G41.8, G43.0, G43.1, G43.3, G43.8, G43.9, G44.0, G44.2, G44.3, G44.8, G45.02, G45.12, G45.13, G45.42, G45.82, G45.89, G45.99, G46.3, G46.4, G46.8, G47.0, G47.1, G47.2, G47.30, G47.31, G47.38, G47.39, G47.4, G47.8, G47.9, G50.0, G50.1, G51.0, G52.3, G54.0, G54.3, G54.5, G54.6, G54.9, G55.1, G55.2, G55.3, G56.0, G56.1, G56.2, G56.3, G56.8, G56.9, G57.0, G57.1, G57.2, G57.3, G57.4, G57.6, G57.8, G57.9, G58.0, G58.8, G59.0, G60.0, G60.8, G60.9, G61.0, G61.8, G62.0, G62.1, G62.2, G62.80, G62.88, G62.9, G63.0, G63.2, G63.4, G70.0, G70.8, G71.0, G71.1, G71.2, G71.9, G72.4, G72.80, G72.88, G73.4, G80.0, G80.1, G80.2, G80.4, G80.8, G80.9, G81.0, G81.1, G81.9, G82.00, G82.01, G82.02, G82.03, G82.09, G82.11, G82.13, G82.19, G82.20, G82.21, G82.22, G82.23, G82.29, G82.30, G82.31, G82.33, G82.39, G82.40, G82.42, G82.43, G82.49, G82.53, G82.59, G82.61, G82.62, G82.63, G82.64, G82.65, G82.66, G82.67, G82.69, G83.1, G83.2, G83.3, G83.40, G83.41, G83.6, G83.8, G83.9, G90.09, G90.2, G90.51, G90.59, G90.79, G91.0, G91.1, G91.20, G91.21, G91.29, G91.3, G91.8, G91.9, G93.0, G93.1, G93.2, G93.3, G93.4, G93.5, G93.6, G93.80, G93.88, G94.2, G94.3, G94.8, G95.0, G95.10, G95.18, G95.2, G95.80, G95.81, G95.88, G96.0, G96.1, G96.8, G97.1, G97.80, G97.82, G97.9, G99.0, G99.2, H00.0, H00.1, H01.0, H02.0, H02.1, H02.2, H02.4, H02.5, H02.8, H03.1, H04.1, H04.2, H04.5, H05.2, H05.8, H10.0, H10.2, H10.3, H10.5, H10.8, H10.9, H11.3, H11.4, H11.8, H11.9, H13.2, H16.0, H16.2, H18.0, H18.2, H18.4, H18.8, H18.9, H19.2, H19.3, H21.0, H25.1, H25.8, H25.9, H26.0, H26.8, H27.0, H27.1, H28.0, H31.8, H33.0, H33.2, H34.0, H34.1, H35.0, H35.3, H35.30, H35.31, H35.38, H35.39, H35.6, H35.8, H35.9, H36.0, H40.0, H40.1, H40.2, H40.3, H40.8, H40.9, H42.0, H43.1, H43.3, H43.8, H47.0, H47.1, H47.2, H47.3, H49.0, H49.1, H49.2, H49.4, H50.1, H50.4, H50.5, H50.6, H50.9, H51.0, H51.1, H51.2, H51.8, H51.9, H52.1, H53.0, H53.1, H53.2, H53.3, H53.4, H53.8, H53.9, H54.0, H54.1, H54.2, H54.4, H54.9, H55, H57.0, H57.1, H57.8, H57.9, H58.1, H58.8, H59.8, H60.3, H60.5, H60.8, H60.9, H61.0, H61.1, H61.2, H61.9, H65.0, H65.1, H65.2, H65.3, H65.9, H66.0, H66.9, H69.8, H69.9, H72.0, H72.2, H72.8, H72.9, H73.9, H74.8, H81.0, H81.1, H81.2, H81.3, H81.4, H81.8, H81.9, H83.0, H83.3, H83.9, H90.0, H90.1, H90.2, H90.3, H90.4, H90.5, H90.6, H91.0, H91.1, H91.2, H91.3, H91.8, H91.9, H92.0, H92.1, H92.2, H93.0, H93.1, H93.2, H95.0, I01.9, I05.0, I05.1, I05.2, I05.8, I06.1, I07.1, I08.0, I08.1, I08.2, I08.3, I09.9, I10.00, I10.01, I10.90, I10.91, I11.00, I11.01, I11.90, I12.00, I12.01, I13.00, I13.10, I13.20, I15.00, I15.10, I15.21, I15.80, I20.0, I20.1, I20.8, I21.0, I21.1, I21.2, I21.3, I21.4, I21.9, I24.1, I24.9, I25.0, I25.10, I25.11, I25.12, I25.13, I25.14, I25.15, I25.16, I25.19, I25.20, I25.21, I25.22, I25.3, I25.4, I25.5, I25.8, I25.9, I26.0, I26.9, I27.0, I27.20, I27.28, I27.8, I30.9, I31.2, I31.3, I31.9, I33.0, I34.0, I34.2, I34.80, I35.0, I35.1, I35.2, I35.8, I36.0, I36.1, I37.1, I38, I39.1, I39.8, I42.0, I42.1, I42.2, I42.7, I42.88, I42.9, I43.1, I43.8, I44.0, I44.1, I44.2, I44.3, I44.4, I44.6, I44.7, I45.0, I45.1, I45.2, I45.5, I45.6, I46.0, I46.1, I46.9, I47.1, I47.2, I47.9, I48.0, I48.1, I48.2, I48.3, I48.4, I48.9, I49.0, I49.3, I49.4, I49.5, I49.8, I49.9, I50.01, I50.02, I50.03, I50.04, I50.05, I50.11, I50.12, I50.13, I50.14, I50.19, I50.9, I51.3, I51.7, I51.9, I60.0, I60.1, I60.2, I60.3, I60.4, I60.7, I60.8, I60.9, I61.0, I61.1, I61.2, I61.3, I61.4, I61.5, I61.6, I61.8, I61.9, I62.00, I62.01, I62.02, I62.09, I62.9, I63.0, I63.1, I63.2, I63.3, I63.4, I63.5, I63.8, I63.9, I64, I65.0, I65.1, I65.2, I65.3, I65.8, I66.0, I66.1, I66.4, I66.8, I67.0, I67.10, I67.11, I67.3, I67.4, I67.5, I67.6, I67.80, I67.88, I68.0, I69.0, I69.1, I69.2, I69.3, I69.4, I70.0, I70.1, I70.20, I70.21, I70.22, I70.23, I70.24, I70.25, I70.26, I70.29, I70.9, I71.00, I71.01, I71.03, I71.05, I71.1, I71.2, I71.3, I71.4, I71.5, I71.6, I71.9, I72.0, I72.1, I72.2, I72.3, I72.4, I72.5, I72.6, I72.8, I72.9, I73.0, I73.8, I73.9, I74.0, I74.2, I74.3, I74.5, I74.8, I77.0, I77.1, I77.2, I77.6, I77.9, I79.2, I80.0, I80.1, I80.20, I80.28, I80.3, I80.80, I80.81, I80.88, I80.9, I81, I82.2, I82.3, I82.80, I82.81, I82.88, I82.9, I83.0, I83.1, I83.2, I83.9, I85.9, I86.1, I86.82, I86.88, I87.00, I87.01, I87.1, I87.20, I87.21, I88.0, I88.1, I88.8, I88.9, I89.00, I89.01, I89.08, I89.09, I89.1, I89.8, I95.0, I95.1, I95.2, I95.8, I95.9, I97.80, I97.85, I97.87, I97.88, I97.89, I98.2, I99, J00, J01.0, J01.3, J01.8, J01.9, J02.8, J02.9, J03.0, J03.8, J03.9, J04.0, J04.2, J05.1, J06.0, J06.8, J06.9, J10.0, J10.1, J10.8, J11.1, J11.8, J12.8, J12.9, J13, J14, J15.0, J15.1, J15.2, J15.3, J15.4, J15.5, J15.6, J15.7, J15.8, J15.9, J16.0, J16.8, J17.0, J17.2, J17.8, J18.0, J18.1, J18.2, J18.8, J18.9, J20.2, J20.6, J20.8, J20.9, J21.0, J22, J30.0, J30.1, J30.3, J32.0, J32.4, J34.2, J34.3, J34.8, J35.2, J38.00, J38.01, J38.02, J38.3, J38.4, J38.5, J38.6, J39.0, J39.2, J39.88, J40, J41.0, J41.1, J42, J43.0, J43.1, J43.2, J43.8, J43.9, J44.00, J44.01, J44.02, J44.03, J44.09, J44.10, J44.11, J44.12, J44.13, J44.19, J44.80, J44.81, J44.82, J44.83, J44.89, J44.92, J44.99, J45.0, J45.00, J45.09, J45.1, J45.10, J45.19, J45.8, J45.89, J45.9, J45.90, J45.99, J47, J63.4, J64, J69.0, J69.8, J80.01, J80.02, J80.03, J80.09, J81, J82, J84.1, J84.90, J85.1, J85.2, J86.0, J86.9, J90, J91, J93.0, J93.1, J93.8, J93.9, J94.1, J94.2, J94.8, J94.9, J95.0, J95.1, J95.2, J95.4, J95.5, J95.80, J95.88, J96.00, J96.01, J96.09, J96.10, J96.11, J96.19, J96.90, J96.91, J96.99, J98.0, J98.1, J98.10, J98.11, J98.12, J98.18, J98.2, J98.4, J98.50, J98.6, J98.7, J98.8, J99.1, J99.23, J99.8, K01.0, K02.1, K02.8, K02.9, K03.0, K03.1, K04.5, K04.8, K04.9, K05.1, K05.2, K05.3, K05.4, K05.5, K06.2, K07.2, K07.3, K07.6, K08.0, K08.1, K08.3, K08.81, K08.88, K08.9, K09.0, K09.2, K10.28, K10.8, K11.2, K11.4, K11.5, K11.7, K12.0, K12.3, K13.0, K13.1, K13.2, K13.7, K14.8, K20, K20.1, K21.0, K21.9, K22.0, K22.1, K22.2, K22.3, K22.7, K22.81, K22.88, K23.8, K25.0, K25.2, K25.3, K25.4, K25.5, K25.7, K25.9, K26.0, K26.1, K26.3, K26.4, K26.7, K29.0, K29.1, K29.3, K29.4, K29.5, K29.6, K29.7, K29.8, K29.9, K31.6, K31.88, K31.9, K35.31, K35.8, K40.00, K40.20, K40.90, K40.91, K41.90, K41.91, K42.0, K42.9, K43.1, K43.2, K43.60, K43.90, K43.98, K43.99, K44.9, K45.0, K50.0, K50.1, K50.82, K50.88, K50.9, K51.0, K51.5, K51.8, K51.9, K52.0, K52.1, K52.9, K55.0, K55.1, K55.21, K55.9, K56.0, K56.4, K56.5, K56.6, K57.22, K57.30, K57.31, K57.32, K57.92, K58.8, K58.9, K59.0, K59.00, K59.01, K59.02, K59.09, K59.1, K59.2, K59.3, K59.8, K59.9, K60.0, K60.2, K60.4, K61.0, K62.2, K62.3, K62.5, K62.6, K62.8, K62.9, K63.1, K63.2, K63.5, K63.8, K64.0, K64.1, K64.2, K64.4, K64.5, K65.0, K65.00, K65.09, K65.8, K66.0, K66.1, K67.8, K70.0, K70.2, K70.3, K70.4, K70.41, K71.0, K71.1, K71.2, K71.6, K72.0, K72.1, K72.71, K72.72, K72.74, K72.79, K74.0, K74.3, K74.6, K74.70, K74.71, K74.72, K75.0, K75.4, K75.8, K76.0, K76.1, K76.2, K76.3, K76.6, K76.7, K76.8, K76.9, K80.00, K80.01, K80.10, K80.20, K80.30, K80.31, K80.40, K80.51, K80.80, K81.0, K81.1, K81.9, K82.1, K82.2, K82.8, K83.0, K83.08, K83.1, K83.3, K83.8, K85.00, K85.01, K85.10, K85.80, K85.81, K85.90, K86.0, K86.1, K86.18, K86.2, K86.8, K86.83, K86.88, K86.9, K90.0, K91.2, K91.3, K91.4, K91.83, K91.88, K92.0, K92.1, K92.2, L01.0, L01.1, L02.0, L02.1, L02.2, L02.3, L02.4, L02.8, L02.9, L03.01, L03.02, L03.10, L03.11, L03.2, L03.3, L03.9, L05.0, L05.9, L08.0, L08.1, L08.8, L08.9, L10.9, L12.0, L20.0, L20.8, L20.9, L21.0, L21.8, L21.9, L22, L23.0, L23.1, L23.2, L23.3, L23.5, L23.9, L24.0, L24.9, L25.9, L27.0, L27.1, L27.8, L28.1, L28.2, L29.8, L29.9, L30.0, L30.3, L30.4, L30.8, L30.9, L40.0, L40.3, L40.5, L40.8, L40.9, L44.8, L50.0, L50.2, L50.6, L50.8, L50.9, L52, L53.0, L53.8, L53.9, L57.0, L58.0, L60.0, L60.1, L60.2, L60.8, L60.9, L70.2, L71.0, L71.8, L72.1, L72.9, L73.2, L73.8, L73.9, L74.1, L81.1, L82, L84, L85.3, L88, L89.00, L89.02, L89.03, L89.04, L89.05, L89.07, L89.08, L89.09, L89.10, L89.11, L89.12, L89.13, L89.14, L89.15, L89.16, L89.17, L89.18, L89.19, L89.20, L89.21, L89.22, L89.24, L89.25, L89.26, L89.27, L89.28, L89.29, L89.30, L89.33, L89.34, L89.35, L89.36, L89.37, L89.91, L89.92, L89.94, L89.97, L89.98, L89.99, L90.5, L90.8, L91.0, L91.8, L92.0, L92.3, L93.0, L93.1, L97, L98.1, L98.4, L98.7, L98.8, L98.9, L99.11, L99.23, L99.8, M00.00, M00.01, M00.02, M00.03, M00.04, M00.05, M00.06, M00.07, M00.09, M00.15, M00.16, M00.17, M00.25, M00.26, M00.27, M00.81, M00.82, M00.85, M00.86, M00.87, M00.90, M00.91, M00.92, M00.94, M00.95, M00.96, M00.97, M00.99, M01.31, M02.00, M02.33, M02.39, M02.80, M02.86, M02.90, M02.96, M02.97, M05.30, M05.39, M05.80, M05.89, M05.90, M05.99, M06.00, M06.01, M06.05, M06.09, M06.37, M06.40, M06.44, M06.46, M06.48, M06.49, M06.80, M06.86, M06.89, M06.90, M06.94, M06.96, M06.99, M07.30, M07.39, M08.40, M08.46, M08.80, M08.85, M09.09, M10.00, M10.01, M10.02, M10.03, M10.04, M10.05, M10.06, M10.07, M10.09, M10.37, M10.40, M10.43, M10.46, M10.90, M10.91, M10.94, M10.97, M10.98, M10.99, M11.26, M11.29, M12.02, M12.26, M12.32, M12.57, M12.80, M12.85, M12.86, M13.0, M13.15, M13.16, M13.17, M13.80, M13.81, M13.87, M13.90, M13.96, M14.27, M14.67, M14.69, M14.80, M15.0, M15.8, M15.9, M16.0, M16.1, M16.2, M16.3, M16.4, M16.5, M16.6, M16.7, M16.9, M17.0, M17.1, M17.2, M17.3, M17.4, M17.5, M17.9, M18.0, M18.1, M18.9, M19.01, M19.02, M19.03, M19.04, M19.05, M19.07, M19.08, M19.09, M19.14, M19.17, M19.21, M19.22, M19.27, M19.81, M19.84, M19.85, M19.87, M19.89, M19.91, M19.94, M19.97, M19.98, M19.99, M20.1, M20.2, M20.5, M20.6, M21.00, M21.05, M21.06, M21.15, M21.16, M21.30, M21.33, M21.37, M21.4, M21.50, M21.57, M21.61, M21.62, M21.68, M21.76, M21.80, M21.81, M21.85, M21.86, M21.99, M22.0, M22.1, M22.2, M22.3, M22.4, M22.9, M23.00, M23.03, M23.13, M23.16, M23.20, M23.21, M23.23, M23.26, M23.29, M23.30, M23.31, M23.32, M23.33, M23.34, M23.35, M23.36, M23.39, M23.4, M23.50, M23.51, M23.59, M23.60, M23.61, M23.62, M23.63, M23.64, M23.69, M23.80, M23.81, M23.83, M23.89, M23.90, M23.91, M23.93, M23.95, M23.96, M23.99, M24.00, M24.02, M24.05, M24.07, M24.10, M24.11, M24.12, M24.14, M24.17, M24.21, M24.24, M24.27, M24.30, M24.33, M24.34, M24.35, M24.36, M24.37, M24.40, M24.41, M24.42, M24.43, M24.44, M24.45, M24.47, M24.50, M24.55, M24.56, M24.63, M24.65, M24.66, M24.69, M24.7, M24.82, M24.85, M24.87, M24.89, M24.90, M24.91, M24.92, M24.95, M24.99, M25.00, M25.06, M25.15, M25.16, M25.17, M25.20, M25.21, M25.25, M25.26, M25.30, M25.31, M25.32, M25.33, M25.34, M25.35, M25.40, M25.42, M25.45, M25.46, M25.47, M25.50, M25.51, M25.52, M25.53, M25.54, M25.55, M25.56, M25.57, M25.58, M25.59, M25.61, M25.62, M25.65, M25.66, M25.70, M25.73, M25.75, M25.77, M25.78, M25.81, M25.85, M25.86, M25.90, M25.91, M25.95, M25.96, M30.3, M31.3, M31.4, M31.6, M31.7, M32.1, M32.8, M32.9, M33.1, M34.0, M34.1, M35.0, M35.2, M35.3, M35.6, M35.9, M40.00, M40.02, M40.05, M40.14, M40.15, M40.16, M40.22, M40.24, M40.25, M40.29, M41.00, M41.20, M41.24, M41.26, M41.40, M41.59, M41.84, M41.85, M41.86, M41.90, M41.94, M41.96, M41.99, M42.00, M42.09, M42.10, M42.12, M42.13, M42.14, M42.15, M42.16, M42.17, M42.19, M42.90, M42.92, M42.96, M42.97, M42.99, M43.00, M43.02, M43.06, M43.10, M43.12, M43.13, M43.15, M43.16, M43.17, M43.29, M43.3, M43.6, M43.87, M43.93, M43.94, M43.95, M43.99, M45.00, M45.06, M45.07, M45.08, M45.09, M46.00, M46.06, M46.1, M46.20, M46.22, M46.23, M46.25, M46.27, M46.28, M46.32, M46.34, M46.39, M46.40, M46.42, M46.43, M46.44, M46.45, M46.46, M46.47, M46.48, M46.49, M46.57, M46.59, M46.81, M46.86, M46.93, M46.96, M46.97, M46.99, M47.12, M47.15, M47.21, M47.22, M47.23, M47.26, M47.27, M47.29, M47.80, M47.82, M47.84, M47.85, M47.86, M47.87, M47.88, M47.89, M47.92, M47.93, M47.95, M47.96, M47.97, M47.99, M48.00, M48.02, M48.03, M48.04, M48.05, M48.06, M48.07, M48.08, M48.09, M48.10, M48.12, M48.36, M48.40, M48.44, M48.45, M48.46, M48.48, M48.52, M48.54, M48.55, M48.56, M48.58, M48.59, M48.86, M49.05, M49.49, M49.50, M49.52, M49.53, M49.54, M49.55, M49.56, M49.58, M49.59, M50.0, M50.1, M50.2, M50.8, M50.9, M51.0, M51.1, M51.2, M51.3, M51.8, M51.9, M53.0, M53.1, M53.20, M53.21, M53.22, M53.24, M53.25, M53.26, M53.27, M53.28, M53.29, M53.3, M53.82, M53.83, M53.85, M53.90, M53.92, M53.99, M54.00, M54.01, M54.02, M54.04, M54.09, M54.10, M54.11, M54.12, M54.13, M54.14, M54.15, M54.16, M54.17, M54.18, M54.19, M54.2, M54.3, M54.4, M54.5, M54.6, M54.80, M54.81, M54.82, M54.83, M54.84, M54.85, M54.86, M54.87, M54.88, M54.89, M54.90, M54.92, M54.94, M54.95, M54.96, M54.97, M54.98, M54.99, M60.00, M60.01, M60.02, M60.05, M60.06, M60.07, M60.08, M60.16, M60.26, M60.27, M60.88, M62.00, M62.01, M62.02, M62.06, M62.08, M62.10, M62.11, M62.12, M62.15, M62.16, M62.18, M62.20, M62.22, M62.23, M62.24, M62.25, M62.26, M62.27, M62.28, M62.30, M62.33, M62.37, M62.40, M62.41, M62.42, M62.45, M62.48, M62.49, M62.50, M62.52, M62.55, M62.59, M62.60, M62.61, M62.62, M62.63, M62.64, M62.65, M62.66, M62.68, M62.69, M62.80, M62.81, M62.82, M62.84, M62.85, M62.86, M62.88, M62.89, M62.90, M62.91, M62.98, M62.99, M65.00, M65.01, M65.06, M65.10, M65.11, M65.13, M65.14, M65.15, M65.16, M65.17, M65.20, M65.22, M65.23, M65.25, M65.26, M65.27, M65.28, M65.29, M65.3, M65.4, M65.80, M65.83, M65.84, M65.85, M65.86, M65.87, M65.89, M65.90, M65.91, M65.92, M65.93, M65.94, M65.95, M65.96, M65.97, M65.99, M66.0, M66.10, M66.15, M66.23, M66.36, M66.37, M67.0, M67.17, M67.36, M67.40, M67.41, M67.43, M67.44, M67.45, M67.48, M67.49, M67.81, M67.84, M67.86, M67.99, M68.81, M70.0, M70.1, M70.2, M70.3, M70.4, M70.5, M70.6, M70.7, M70.8, M70.9, M71.00, M71.01, M71.02, M71.03, M71.05, M71.06, M71.12, M71.15, M71.16, M71.17, M71.2, M71.36, M71.40, M71.42, M71.50, M71.56, M71.57, M71.58, M71.85, M71.86, M71.92, M71.99, M72.0, M72.2, M72.40, M72.60, M72.66, M72.68, M72.85, M72.86, M72.87, M73.86, M75.0, M75.1, M75.2, M75.3, M75.4, M75.5, M75.6, M75.8, M76.0, M76.1, M76.3, M76.5, M76.6, M76.7, M76.8, M76.9, M77.0, M77.1, M77.2, M77.3, M77.4, M77.5, M77.8, M77.9, M79.00, M79.01, M79.04, M79.06, M79.09, M79.10, M79.11, M79.12, M79.13, M79.15, M79.16, M79.17, M79.18, M79.19, M79.20, M79.21, M79.23, M79.26, M79.27, M79.28, M79.29, M79.30, M79.33, M79.46, M79.50, M79.51, M79.52, M79.53, M79.54, M79.55, M79.56, M79.57, M79.58, M79.60, M79.61, M79.62, M79.63, M79.64, M79.65, M79.66, M79.67, M79.69, M79.70, M79.80, M79.81, M79.82, M79.83, M79.84, M79.85, M79.86, M79.87, M79.88, M79.89, M79.91, M79.92, M79.93, M79.96, M79.97, M79.99, M80.00, M80.02, M80.05, M80.08, M80.09, M80.18, M80.20, M80.25, M80.48, M80.55, M80.58, M80.85, M80.88, M80.98, M81.00, M81.01, M81.05, M81.06, M81.08, M81.09, M81.20, M81.25, M81.40, M81.48, M81.50, M81.80, M81.85, M81.88, M81.89, M81.90, M81.98, M81.99, M82.00, M83.10, M83.39, M84.00, M84.01, M84.02, M84.03, M84.06, M84.07, M84.08, M84.11, M84.12, M84.13, M84.15, M84.16, M84.17, M84.18, M84.26, M84.27, M84.31, M84.35, M84.37, M84.39, M84.40, M84.42, M84.45, M84.46, M84.48, M84.80, M84.85, M84.97, M85.00, M85.42, M85.45, M85.48, M85.52, M85.55, M85.56, M85.62, M85.65, M85.68, M85.80, M85.84, M85.85, M85.88, M85.89, M86.00, M86.01, M86.02, M86.03, M86.05, M86.06, M86.07, M86.11, M86.12, M86.15, M86.16, M86.17, M86.21, M86.22, M86.23, M86.25, M86.26, M86.27, M86.30, M86.35, M86.36, M86.41, M86.45, M86.46, M86.47, M86.55, M86.57, M86.61, M86.62, M86.64, M86.65, M86.66, M86.67, M86.69, M86.81, M86.82, M86.85, M86.86, M86.87, M86.90, M86.92, M86.95, M86.97, M86.99, M87.00, M87.05, M87.06, M87.25, M87.26, M87.35, M87.38, M87.85, M87.86, M87.95, M87.96, M88.80, M88.81, M89.00, M89.01, M89.30, M89.50, M89.52, M89.55, M89.58, M89.82, M89.85, M89.86, M89.87, M89.88, M89.95, M90.11, M90.70, M90.71, M90.72, M90.75, M90.76, M90.78, M90.79, M90.89, M91.0, M91.1, M91.2, M91.8, M92.5, M92.6, M92.7, M93.0, M93.1, M93.20, M93.23, M93.25, M93.26, M93.27, M93.29, M93.86, M93.89, M93.9, M94.0, M94.26, M95.1, M95.4, M95.8, M96.0, M96.1, M96.6, M96.82, M96.88, M99.00, M99.43, M99.50, M99.53, M99.63, M99.73, M99.79, M99.80, M99.81, M99.82, M99.83, M99.84, M99.85, M99.87, M99.88, M99.89, M99.94, N02.0, N02.8, N03.2, N04.1, N05.8, N08.3, N08.5, N08.8, N10, N11.1, N13.0, N13.1, N13.2, N13.20, N13.21, N13.29, N13.3, N13.4, N13.5, N13.6, N13.62, N13.65, N13.9, N14.1, N17.09, N17.81, N17.82, N17.83, N17.89, N17.91, N17.92, N17.93, N17.99, N18.1, N18.2, N18.3, N18.4, N18.5, N18.89, N18.9, N20.0, N20.1, N20.2, N20.9, N21.0, N23, N25.0, N25.8, N26, N27.0, N28.0, N28.1, N28.8, N28.88, N29.8, N30.0, N30.2, N30.4, N30.8, N31.0, N31.2, N31.88, N31.9, N32.0, N32.8, N32.9, N34.2, N35.8, N36.0, N36.8, N39.0, N39.3, N39.40, N39.41, N39.42, N39.48, N39.88, N40, N41.0, N41.1, N42.1, N43.2, N44.0, N45.9, N47, N48.2, N48.4, N48.5, N48.8, N50.1, N50.8, N50.9, N70.0, N70.1, N72, N76.0, N76.2, N76.4, N77.1, N80.1, N80.5, N81.1, N81.3, N83.2, N84.1, N85.0, N89.8, N92.0, N93.8, N93.9, N94.5, N94.8, N95.0, N99.0, N99.5, N99.8, N99.9, O03.3, O09.0, O09.1, O09.2, O09.3, O09.4, O09.5, O09.6, O09.9, O21.9, O23.4, O23.5, O23.9, O24.4, O30.0, O33.5, O34.2, O36.4, O36.6, O36.8, O41.1, O44.00, O46.8, O47.0, O60.0, O68.0, O70.1, O80, O82, O99.0, O99.2, O99.3, O99.4, O99.5, O99.6, O99.8, P12.0, P50.2, P96.0, Q02, Q03.0, Q03.9, Q04.0, Q04.6, Q04.8, Q05.2, Q07.0, Q20.3, Q21.0, Q21.1, Q21.3, Q23.1, Q23.2, Q24.5, Q27.3, Q28.28, Q30.8, Q34.8, Q61.0, Q61.2, Q61.9, Q65.1, Q65.5, Q65.6, Q65.8, Q65.9, Q66.0, Q66.5, Q66.8, Q66.9, Q67.4, Q68.2, Q74.0, Q74.1, Q74.2, Q74.8, Q75.3, Q76.21, Q76.4, Q78.0, Q78.4, Q79.6, Q82.2, Q82.5, Q82.8, Q82.9, Q85.0, Q85.8, Q87.2, Q87.4, Q87.8, Q90.0, Q90.1, Q90.2, Q90.9, Q92.1, Q93.4, Q96.9, Q98.0, R00.0, R00.1, R00.2, R00.3, R01.2, R02.00, R02.01, R02.02, R02.03, R02.04, R02.05, R02.06, R02.07, R02.09, R02.8, R03.0, R03.1, R04.0, R04.1, R04.2, R04.8, R05, R06.0, R06.4, R06.5, R06.88, R07.0, R07.1, R07.2, R07.3, R07.4, R09.0, R09.1, R09.2, R10.0, R10.1, R10.2, R10.3, R10.4, R11, R12, R13.0, R13.1, R13.9, R14, R15, R16.0, R16.1, R16.2, R17.9, R18, R19.0, R19.5, R19.80, R20.0, R20.1, R20.2, R20.8, R21, R22.0, R22.1, R22.3, R22.4, R22.9, R23.0, R23.1, R23.3, R23.8, R25.1, R25.2, R25.3, R25.8, R26.0, R26.1, R26.2, R26.3, R26.8, R27.0, R27.8, R29.2, R29.5, R29.6, R29.8, R30.0, R31, R32, R33, R34, R35, R35.0, R35.1, R39.0, R39.1, R39.8, R40.0, R40.1, R40.2, R41.0, R41.1, R41.2, R41.3, R41.8, R42, R43.0, R43.2, R44.0, R44.1, R44.3, R45.1, R45.4, R45.5, R45.6, R45.8, R46.0, R46.2, R46.3, R46.4, R47.0, R47.1, R47.8, R48.1, R48.2, R49.0, R49.1, R49.8, R50.2, R50.80, R50.88, R50.9, R51, R52.0, R52.1, R52.2, R52.9, R53, R55, R56.0, R56.8, R57.0, R57.1, R57.2, R57.8, R57.9, R58, R59.0, R59.9, R60.0, R60.1, R60.9, R61.9, R62.0, R63.0, R63.1, R63.3, R63.4, R63.5, R63.6, R63.8, R64, R65.0, R65.1, R65.2, R65.3, R68.0, R68.8, R69, R72, R73.9, R74.0, R74.8, R77.88, R79.8, R79.9, R82.1, R82.4, R85.8, R90.0, R90.8, R91, R93.0, R93.3, R93.5, R93.6, R94.0, R94.1, R94.2, R94.3, R99, S00.00, S00.01, S00.02, S00.03, S00.04, S00.05, S00.08, S00.1, S00.20, S00.21, S00.24, S00.28, S00.30, S00.31, S00.33, S00.34, S00.35, S00.38, S00.40, S00.41, S00.43, S00.44, S00.45, S00.48, S00.50, S00.51, S00.54, S00.55, S00.58, S00.7, S00.80, S00.81, S00.83, S00.84, S00.85, S00.88, S00.90, S00.91, S00.92, S00.93, S00.95, S00.98, S01.0, S01.1, S01.20, S01.21, S01.22, S01.29, S01.30, S01.31, S01.33, S01.34, S01.37, S01.39, S01.41, S01.42, S01.43, S01.49, S01.50, S01.51, S01.52, S01.53, S01.54, S01.55, S01.59, S01.7, S01.80, S01.83, S01.84, S01.85, S01.86, S01.87, S01.88, S01.89, S01.9, S02.0, S02.1, S02.2, S02.3, S02.4, S02.49, S02.5, S02.60, S02.61, S02.62, S02.63, S02.64, S02.65, S02.66, S02.67, S02.68, S02.69, S02.7, S02.8, S02.9, S03.0, S03.1, S03.2, S03.3, S03.4, S03.5, S04.0, S04.1, S04.2, S04.3, S04.4, S04.5, S04.7, S04.8, S05.0, S05.1, S05.2, S05.3, S05.4, S05.5, S05.6, S05.7, S05.8, S05.9, S06.0, S06.1, S06.20, S06.21, S06.23, S06.28, S06.30, S06.31, S06.32, S06.33, S06.34, S06.38, S06.4, S06.5, S06.6, S06.70, S06.71, S06.72, S06.73, S06.79, S06.8, S06.9, S07.0, S07.9, S08.0, S08.1, S08.8, S09.0, S09.1, S09.2, S09.7, S09.8, S09.9, S10.0, S10.18, S10.7, S10.80, S10.81, S10.84, S10.85, S10.88, S10.90, S10.95, S10.98, S11.01, S11.02, S11.1, S11.7, S11.80, S11.84, S11.85, S11.86, S11.87, S11.88, S11.89, S11.9, S12.0, S12.1, S12.21, S12.22, S12.23, S12.24, S12.25, S12.7, S12.8, S12.9, S13.0, S13.11, S13.12, S13.14, S13.15, S13.16, S13.17, S13.2, S13.3, S13.4, S13.5, S13.6, S14.0, S14.10, S14.11, S14.12, S14.13, S14.2, S14.3, S14.4, S14.5, S14.70, S14.71, S14.72, S14.73, S14.74, S14.75, S14.76, S14.77, S15.00, S15.01, S15.02, S15.03, S15.1, S15.2, S15.3, S15.7, S15.88, S16, S17.0, S19.80, S19.88, S19.9, S20.0, S20.10, S20.11, S20.14, S20.2, S20.30, S20.31, S20.40, S20.41, S20.42, S20.48, S20.7, S20.80, S20.81, S20.85, S20.88, S21.0, S21.1, S21.2, S21.7, S21.80, S21.83, S21.84, S21.85, S21.86, S21.87, S21.88, S21.89, S21.9, S22.00, S22.01, S22.02, S22.03, S22.04, S22.05, S22.06, S22.1, S22.2, S22.20, S22.21, S22.22, S22.23, S22.31, S22.32, S22.40, S22.41, S22.42, S22.43, S22.44, S22.5, S22.8, S22.9, S23.0, S23.10, S23.17, S23.2, S23.3, S23.4, S23.5, S24.0, S24.10, S24.11, S24.12, S24.2, S24.5, S24.70, S24.72, S24.73, S24.74, S24.75, S24.76, S24.77, S25.0, S25.1, S25.2, S25.5, S25.7, S25.80, S25.88, S26.0, S26.81, S26.83, S26.88, S27.0, S27.1, S27.2, S27.31, S27.32, S27.38, S27.4, S27.5, S27.6, S27.7, S27.81, S27.83, S27.88, S29.0, S29.80, S29.88, S29.9, S30.0, S30.1, S30.2, S30.7, S30.80, S30.81, S30.82, S30.84, S30.85, S30.86, S30.9, S30.91, S30.98, S31.0, S31.1, S31.2, S31.3, S31.4, S31.7, S31.80, S31.83, S31.84, S31.85, S31.86, S31.87, S31.88, S31.89, S32.00, S32.01, S32.02, S32.03, S32.04, S32.05, S32.1, S32.2, S32.3, S32.4, S32.5, S32.7, S32.81, S32.82, S32.83, S32.89, S33.0, S33.15, S33.2, S33.3, S33.4, S33.50, S33.6, S33.7, S34.0, S34.11, S34.18, S34.2, S34.30, S34.31, S34.38, S34.4, S34.70, S34.71, S34.72, S34.73, S34.74, S34.77, S35.0, S35.1, S35.2, S35.3, S35.4, S35.5, S35.7, S35.88, S35.9, S36.00, S36.01, S36.02, S36.03, S36.04, S36.08, S36.11, S36.12, S36.13, S36.14, S36.15, S36.16, S36.17, S36.18, S36.20, S36.21, S36.22, S36.23, S36.29, S36.3, S36.40, S36.41, S36.49, S36.50, S36.51, S36.52, S36.53, S36.54, S36.59, S36.6, S36.7, S36.81, S36.82, S36.83, S36.88, S37.00, S37.01, S37.02, S37.03, S37.1, S37.20, S37.21, S37.22, S37.28, S37.30, S37.31, S37.38, S37.81, S37.88, S37.9, S38.0, S38.1, S38.2, S38.3, S39.0, S39.6, S39.7, S39.80, S39.81, S39.88, S39.9, S40.0, S40.7, S40.81, S40.84, S40.86, S40.88, S40.9, S41.0, S41.1, S41.80, S41.84, S41.85, S41.86, S41.87, S41.88, S41.89, S42.00, S42.01, S42.02, S42.03, S42.09, S42.10, S42.11, S42.12, S42.13, S42.14, S42.19, S42.20, S42.21, S42.22, S42.23, S42.24, S42.29, S42.3, S42.40, S42.41, S42.42, S42.43, S42.44, S42.45, S42.49, S42.7, S42.8, S42.9, S43.00, S43.01, S43.02, S43.03, S43.08, S43.1, S43.2, S43.3, S43.4, S43.5, S43.6, S43.7, S44.0, S44.1, S44.2, S44.3, S44.9, S45.0, S45.1, S45.2, S45.3, S45.7, S45.8, S45.9, S46.0, S46.1, S46.2, S46.3, S46.8, S46.9, S47, S48.0, S49.7, S49.8, S49.9, S50.0, S50.1, S50.7, S50.81, S50.82, S50.84, S50.86, S50.88, S50.9, S51.0, S51.7, S51.80, S51.84, S51.85, S51.86, S51.87, S51.88, S51.89, S51.9, S52.00, S52.01, S52.02, S52.09, S52.10, S52.11, S52.12, S52.19, S52.20, S52.21, S52.30, S52.31, S52.4, S52.50, S52.51, S52.52, S52.59, S52.6, S52.7, S52.8, S52.9, S53.0, S53.10, S53.11, S53.12, S53.13, S53.18, S53.2, S53.3, S53.40, S53.41, S53.43, S53.44, S53.48, S54.0, S54.1, S54.2, S54.3, S55.0, S55.1, S55.2, S55.8, S55.9, S56.0, S56.1, S56.2, S56.3, S56.4, S56.5, S56.7, S56.8, S57.0, S57.8, S58.0, S58.1, S59.7, S59.8, S59.9, S60.0, S60.1, S60.2, S60.7, S60.81, S60.82, S60.84, S60.86, S60.88, S60.9, S61.0, S61.1, S61.7, S61.80, S61.84, S61.85, S61.86, S61.87, S61.88, S61.89, S61.9, S62.0, S62.10, S62.11, S62.12, S62.13, S62.14, S62.15, S62.16, S62.17, S62.19, S62.20, S62.21, S62.22, S62.30, S62.31, S62.32, S62.33, S62.34, S62.4, S62.50, S62.51, S62.52, S62.60, S62.61, S62.62, S62.63, S62.7, S62.8, S63.00, S63.01, S63.02, S63.03, S63.04, S63.08, S63.10, S63.11, S63.12, S63.2, S63.3, S63.4, S63.50, S63.52, S63.58, S63.60, S63.61, S63.62, S63.68, S63.7, S64.0, S64.1, S64.2, S64.3, S64.4, S64.8, S65.1, S65.2, S65.4, S65.5, S65.8, S65.9, S66.0, S66.1, S66.2, S66.3, S66.4, S66.6, S66.7, S66.8, S66.9, S67.0, S67.8, S68.0, S68.1, S68.4, S69.8, S70.0, S70.1, S70.7, S70.81, S70.82, S70.84, S70.86, S70.88, S70.9, S71.0, S71.1, S71.7, S71.80, S71.84, S71.85, S71.86, S71.87, S71.88, S71.89, S72.00, S72.01, S72.02, S72.03, S72.04, S72.05, S72.08, S72.10, S72.11, S72.2, S72.3, S72.40, S72.41, S72.42, S72.43, S72.44, S72.7, S72.8, S72.9, S73.00, S73.01, S73.02, S73.08, S73.10, S73.18, S74.0, S74.1, S74.2, S75.0, S75.1, S75.8, S75.9, S76.0, S76.1, S76.2, S76.3, S76.4, S76.7, S77.2, S78.1, S79.7, S79.8, S80.0, S80.1, S80.7, S80.81, S80.82, S80.84, S80.86, S80.88, S80.9, S81.0, S81.7, S81.80, S81.84, S81.85, S81.86, S81.87, S81.88, S81.89, S81.9, S82.0, S82.11, S82.18, S82.21, S82.28, S82.31, S82.38, S82.40, S82.41, S82.42, S82.49, S82.5, S82.6, S82.7, S82.81, S82.82, S82.88, S82.9, S83.0, S83.10, S83.11, S83.13, S83.14, S83.18, S83.2, S83.3, S83.40, S83.41, S83.42, S83.43, S83.44, S83.50, S83.51, S83.52, S83.53, S83.54, S83.6, S83.7, S84.0, S84.1, S84.8, S85.0, S85.1, S85.2, S85.3, S85.5, S85.7, S85.8, S86.0, S86.1, S86.2, S86.3, S86.7, S86.8, S86.9, S87.0, S87.8, S88.0, S88.1, S88.9, S89.8, S89.9, S90.0, S90.1, S90.2, S90.3, S90.7, S90.81, S90.82, S90.84, S90.86, S90.88, S90.9, S91.0, S91.1, S91.2, S91.3, S91.7, S91.80, S91.84, S91.85, S91.86, S91.87, S91.88, S91.89, S92.0, S92.1, S92.20, S92.21, S92.22, S92.23, S92.28, S92.3, S92.4, S92.5, S92.7, S92.9, S93.0, S93.10, S93.11, S93.12, S93.2, S93.30, S93.31, S93.33, S93.34, S93.38, S93.40, S93.41, S93.42, S93.43, S93.48, S93.5, S93.6, S94.0, S94.2, S94.3, S94.9, S95.0, S95.1, S96.0, S96.1, S96.2, S96.7, S96.8, S96.9, S97.0, S97.1, S97.8, S98.1, S98.2, S98.3, S99.7, S99.8, S99.9, T00.0, T00.1, T00.2, T00.3, T00.8, T00.9, T01.0, T01.1, T01.2, T01.3, T01.6, T01.8, T01.9, T02.00, T02.01, T02.10, T02.20, T02.30, T02.41, T02.50, T02.51, T02.61, T02.70, T02.71, T02.80, T02.81, T03.9, T04.8, T06.0, T06.1, T06.2, T06.5, T06.8, T07, T08.0, T08.1, T09.00, T09.05, T09.08, T09.1, T09.2, T09.3, T10.0, T11.01, T11.05, T11.08, T11.1, T11.2, T11.5, T11.9, T12.0, T13.01, T13.04, T13.05, T13.1, T13.4, T13.5, T13.9, T14.00, T14.01, T14.03, T14.04, T14.05, T14.08, T14.1, T14.20, T14.3, T14.4, T14.5, T14.6, T14.8, T14.9, T15.0, T15.1, T15.8, T15.9, T16, T17.1, T17.8, T18.0, T18.1, T18.2, T18.8, T18.9, T19.0, T20.0, T20.1, T20.20, T20.21, T20.3, T20.4, T21.00, T21.22, T21.24, T21.25, T21.29, T21.32, T21.33, T21.34, T21.35, T21.52, T21.62, T21.82, T21.83, T21.84, T21.85, T21.89, T22.00, T22.21, T22.22, T22.31, T22.32, T22.60, T22.62, T22.81, T22.82, T23.0, T23.1, T23.20, T23.21, T23.3, T24.20, T24.21, T24.3, T24.4, T24.5, T25.20, T25.21, T25.3, T26.0, T26.1, T26.6, T26.9, T27.0, T27.1, T27.2, T27.3, T27.7, T29.3, T30.0, T31.00, T31.10, T31.20, T31.30, T31.31, T31.40, T31.66, T31.81, T31.88, T31.97, T32.00, T32.10, T33.0, T39.2, T39.3, T40.1, T40.2, T40.3, T40.5, T40.6, T41.1, T42.4, T43.0, T44.7, T44.8, T44.9, T45.4, T45.5, T46.0, T47.8, T48.3, T50.9, T51.0, T51.1, T51.8, T51.9, T58, T59.0, T59.8, T59.9, T61.0, T63.4, T65.8, T65.9, T66, T68, T70.8, T71, T73.0, T74.1, T74.2, T75.1, T75.4, T76, T78.1, T78.2, T78.3, T78.4, T78.8, T79.0, T79.2, T79.3, T79.4, T79.5, T79.60, T79.61, T79.62, T79.63, T79.68, T79.69, T79.7, T79.8, T79.9, T80.1, T80.2, T80.3, T80.8, T80.9, T81.0, T81.1, T81.2, T81.3, T81.4, T81.5, T81.6, T81.7, T81.8, T81.9, T82.1, T82.2, T82.3, T82.4, T82.5, T82.7, T82.8, T83.0, T83.1, T83.5, T83.8, T84.00, T84.01, T84.04, T84.05, T84.07, T84.08, T84.10, T84.11, T84.12, T84.13, T84.14, T84.15, T84.16, T84.18, T84.20, T84.28, T84.3, T84.4, T84.5, T84.6, T84.7, T84.8, T84.9, T85.0, T85.1, T85.2, T85.51, T85.52, T85.53, T85.6, T85.72, T85.74, T85.78, T85.81, T85.88, T85.9, T86.01, T86.07, T86.10, T86.11, T86.49, T86.50, T86.51, T86.52, T86.59, T86.9, T87.0, T87.3, T87.4, T87.5, T87.6, T88.0, T88.1, T88.2, T88.4, T88.5, T88.6, T88.7, T88.8, T89.00, T89.01, T89.02, T89.03, T90.0, T90.1, T90.2, T90.3, T90.4, T90.5, T90.8, T91.0, T91.2, T91.3, T91.8, T92.2, T92.5, T93.0, T93.1, T93.2, T93.3, T93.5, T93.6, T94.0, T95.1, T97, T98.1, T98.3, U07.1, U07.2, U07.4, U08.9, U11.9, U50.00, U50.01, U50.10, U50.11, U50.20, U50.21, U50.30, U50.31, U50.40, U50.50, U50.51, U51.00, U51.01, U51.02, U51.10, U51.11, U51.12, U51.20, U51.22, U52.0, U52.1, U52.2, U52.3, U60.1, U60.2, U60.3, U60.9, U63.0, U63.1, U63.2, U63.3, U63.4, U63.6, U69.00, U69.01, U69.02, U69.03, U69.04, U69.11, U69.12, U69.13, U69.40, U69.51, U69.52, U69.53, U69.70, U69.72, U69.74, U69.80, U69.81, U69.83, U69.84, U80.00, U80.01, U80.20, U80.21, U80.30, U80.8, U81.20, U81.21, U81.22, U81.23, U81.24, U81.25, U81.26, U81.28, U81.30, U81.31, U81.40, U81.41, U81.42, U81.43, U81.44, U81.48, U81.50, U81.51, U81.6, U81.8, U83, U99.0, V99, W49.9, W64.9, W87.9, X19.9, X49.9, X59.9, X84.9, Y09.9, Y34.9, Y35.7, Y57.9, Y69, Y82.8, Y84.9, Z00.4, Z01.0, Z01.1, Z01.5, Z01.7, Z01.9, Z03.0, Z03.1, Z03.2, Z03.3, Z03.4, Z03.5, Z03.6, Z03.8, Z03.9, Z04.1, Z04.2, Z04.3, Z04.5, Z04.8, Z04.9, Z09.0, Z09.88, Z11, Z12.9, Z13.2, Z13.8, Z20.0, Z20.1, Z20.3, Z20.5, Z20.6, Z20.8, Z20.9, Z21, Z22.1, Z22.3, Z22.5, Z22.8, Z23.5, Z23.6, Z24.2, Z25.8, Z27.3, Z27.8, Z29.0, Z29.1, Z29.21, Z29.28, Z29.8, Z31.4, Z31.6, Z32, Z33, Z34, Z35.0, Z35.1, Z35.8, Z37.0, Z38.0, Z39.1, Z42.0, Z43.0, Z43.1, Z43.2, Z43.3, Z43.4, Z43.5, Z43.6, Z44.8, Z45.00, Z45.01, Z45.20, Z45.88, Z46.6, Z46.7, Z46.8, Z46.9, Z47.0, Z47.8, Z48.0, Z49.0, Z49.1, Z51.1, Z51.4, Z51.5, Z51.83, Z53, Z55, Z59, Z60, Z61, Z62, Z63, Z64.8, Z65, Z72.0, Z72.8, Z73, Z74.0, Z74.1, Z74.2, Z74.3, Z74.8, Z74.9, Z75.67, Z75.78, Z75.8, Z76.4, Z76.8, Z76.9, Z80.0, Z80.1, Z80.3, Z80.9, Z82, Z83.2, Z85.0, Z85.1, Z85.2, Z85.3, Z85.4, Z85.5, Z85.6, Z85.7, Z85.8, Z86.1, Z86.4, Z86.6, Z86.7, Z87.2, Z87.6, Z87.8, Z88.0, Z88.1, Z88.2, Z88.4, Z88.6, Z88.7, Z88.8, Z89.1, Z89.2, Z89.3, Z89.4, Z89.5, Z89.6, Z89.7, Z90.1, Z90.2, Z90.3, Z90.4, Z90.5, Z90.6, Z90.7, Z91.0, Z91.1, Z91.8, Z92.1, Z92.2, Z92.3, Z92.4, Z92.6, Z93.0, Z93.1, Z93.2, Z93.3, Z93.4, Z93.5, Z93.6, Z93.80, Z93.88, Z94.0, Z94.1, Z94.4, Z94.81, Z94.88, Z95.0, Z95.1, Z95.2, Z95.3, Z95.4, Z95.5, Z95.80, Z95.81, Z95.88, Z96.0, Z96.1, Z96.2, Z96.4, Z96.5, Z96.60, Z96.61, Z96.64, Z96.65, Z96.67, Z96.68, Z96.7, Z96.80, Z96.88, Z96.9, Z97.8, Z98.0, Z98.1, Z98.2, Z98.8, Z99.1, Z99.2, Z99.3, Z99.4, Z99.8, Z99.9
